# Not all *Pseudomonas aeruginosa* are equal: strains from industrial sources possess uniquely large multireplicon genomes

**DOI:** 10.1099/mgen.0.000276

**Published:** 2019-06-06

**Authors:** Rebecca Weiser, Angharad E. Green, Matthew J. Bull, Edward Cunningham-Oakes, Keith A. Jolley, Martin C. J. Maiden, Amanda J. Hall, Craig Winstanley, Andrew J. Weightman, Denise Donoghue, Alejandro Amezquita, Thomas R. Connor, Eshwar Mahenthiralingam

**Affiliations:** 1 Microbiomes, Microbes and Informatics Group, Organisms and Environment Division, Cardiff School of Biosciences, Cardiff University, Cardiff, Wales, UK; 2 University of Liverpool, Institute of Infection and Global Health, Liverpool, UK; 3 Department of Zoology, The Tinbergen Building, University of Oxford, Oxford, UK; 4 Unilever Research and Development, Port Sunlight, Wirral, UK; 5 Unilever Research and Development, Safety and Environmental Assurance Centre, Colworth House, Sharnbrook, Bedford, UK

**Keywords:** *Pseudomonas aeruginosa*, industry microbiology, contamination, phylogenomics, megaplasmids

## Abstract

*
Pseudomonas aeruginosa* is a highly versatile, antibiotic-resistant Gram-negative bacterium known for causing opportunistic infections and contamination of industrial products. Despite extensive genomic analysis of clinical *P. aeruginosa* strains, no genomes exist for preservative-tolerant industrial strains. A unique collection of 69 industrial isolates was assembled and compared to clinical and environmental strains; 16 genetically distinct industrial strains were subjected to array tube genotyping, multilocus sequence typing and whole-genome sequencing. The industrial strains possessed high preservative tolerance and were dispersed widely across *P. aeruginosa* as a species, but recurrence of strains from the same lineage within specific industrial products and locations was identified. The industrial *P. aeruginosa* genomes (mean=7.0 Mb) were significantly larger than those of previously sequenced environmental (mean=6.5 Mb; *n*=19) and clinical (mean=6.6 Mb; *n*=66) strains. Complete sequencing of the *P. aeruginosa* industrial strain RW109, which encoded the largest genome (7.75 Mb), revealed a multireplicon structure including a megaplasmid (555 265 bp) and large plasmid (151 612 bp). The RW109 megaplasmid represented an emerging plasmid family conserved in seven industrial and two clinical *P. aeruginosa* strains, and associated with extremely stress-resilient phenotypes, including antimicrobial resistance and solvent tolerance. Here, by defining the detailed phylogenomics of *P. aeruginosa* industrial strains, we show that they uniquely possess multireplicon, megaplasmid-bearing genomes, and significantly greater genomic content worthy of further study.

## Data Summary

All newly determined *Pseudomonas aeruginosa* genome sequences have been deposited at the National Center for Biotechnology Information (NCBI) (BioProject PRJEB8749) with the sequence for strain RW109 (the largest industrial strain in the dataset) available as GCA_900243355.1 (draft) and GCF_900243355.1 (complete). Additional draft *P. aeruginosa* genomes have been deposited as GCA_001374635.1, GCA_001373635.1, GCA_001373875.1, GCA_001374955.1, GCA_001374115.1, GCA_001374355.1, GCA_001374455.1, GCA_001374655.1, GCA_001373655.1, GCA_001373895.1, GCA_001375215.1, GCA_001374135.1, GCA_001374375.1, GCA_001373595.1, GCA_001374675.1, GCA_001374995.1, GCA_001375235.1, GCA_001374155.1, GCA_001374395.1, GCA_001374975.1, GCA_001374435.1, GCA_001373675.1, GCA_001373915.1 and GCA_000568855.1 (full strain information is available in Table S1, available in the online version of this article)

**Impact Statement**

Multiple industrial products are prone to microbial contamination because they are made in a non-sterile way and rely on chemical preservatives to prevent spoilage. However, occasional microbial contamination can result in costly recalls and may pose health risks to consumers. We studied a unique collection of *Pseudomonas aeruginosa* bacteria recovered from industrial products. *P. aeruginosa* can cause infection in vulnerable people and is recognized as an antimicrobial resistance threat, but little is known about industrial strains. We DNA-sequenced the industrial strains and compared their genomes to those of clinical and environmental isolates. Industrial *P. aeruginosa* strains had high preservative tolerance and we found certain strain types reoccurring in the same product type or manufacturing location. Although the industrial strains were genetically diverse in relation to the *P. aeruginosa* population, we uniquely identified that they often carried the same megaplasmid. Megaplasmids are large segments of DNA that can move between bacteria and carry new functions, such as antimicrobial resistance. The megaplasmid, plus other novel genes, gave the industrial *P. aeruginosa* strains very large genomes. What this extra capacity of 500+ genes brings to industrial *P. aeruginosa* in relation to its ability to overcome preservation represents a new challenge in microbiology.

## Introduction

*Pseudomonas aeruginosa* is a widespread human opportunistic pathogen and has been highlighted as a key global threat in relation to antimicrobial resistance, as one of the ESKAPE (* Enterococcus faecium *, *Staphylococcus aureus *, *Klebsiella pneumoniae *, *Acinetobacter baumannii *, *P. aeruginosa* and *Enterobacter cloacae *) pathogens [1]. *P. aeruginosa* is also responsible for chronic lung infections in people with cystic fibrosis (CF) [2], and the pathogenesis, epidemiology, treatment and antibiotic resistance of clinical strains have been major foci of research on the bacterium. However, *P. aeruginosa* is ubiquitous in the environment and can be isolated from diverse habitats, including soil, water and plants [3]. One significant but largely overlooked aspect of *P. aeruginosa *’s biology is its ability to survive in xenobiotic environments, such as industry, where it can contaminate raw materials, pharmaceutical and cosmetic products [4], and even fuels [5].

Within the home and personal care industry, microbial contamination is a major cause of product recalls and may represent a threat to consumer health [6]. *P. aeruginosa* has previously been linked with infections involving contaminated hand lotion [7], mouthwash [8], and make-up products [9]. Industrial manufacturers work to a number of guidelines and International Organization for Standardization (ISO) recommendations to prevent contaminated products from reaching the market place. Preservatives such as parabens, isothiazolinones, formaldehyde releasers, alcohols and organic acids are widely used to maintain product stability and prevent microbial proliferation [10]. When preservation barriers fail, global recall databases show that *P. aeruginosa* is a major cause of contamination, with, for example, >33 % of cosmetic product incidents involving the bacterium [11].

Despite the global reach and billion dollar value of the home and personal care industry, the study of *P. aeruginosa* strains that contaminate industrial products has been limited. What is clear is that these *P. aeruginosa* strains have the ability to overcome the harsh antimicrobial rich environments found in industry and survive to cause product recalls [4]. Understanding the biology of these micro-organisms is therefore important to protect consumers, prevent costly product contamination incidents, improve preservation strategies and assess if they are a reservoir for antimicrobial resistance. Industrial microbiology also represents a relatively unstudied area in terms of the application of genomics, despite microbial contamination impacting on multiple global manufacturing processes. Herein, we have assembled a unique collection of *P. aeruginosa* industrial isolates, contextualized their genotypic and phenotypic characteristics against clinical and environmental strains, and revealed unique insights into their antimicrobial resistance, genome content and organization.

## Methods

### Bacterial collection and phenotypic analysis

*P. aeruginosa* was recovered from 24 contaminated industrial products by serial dilution and growth on selective and non-selective agars as described previously [12]. The 69 industrial isolates were combined with a wider collection of 21 strains from the International *Pseudomonas aeruginosa* Reference Panel [13] (IPARP) and additional reference strains (Table S1). *P. aeruginosa* isolates were stored deep frozen, revived and routinely grown on tryptic soya agar (TSA) or in tryptic soya broth (TSB) (Oxoid Ltd, Basingstoke, UK) as described previously [14]. Multiple databases were analysed for strain information as follows: genome comparison, the *Pseudomonas* genome database [15] and GenBank; random amplified polymorphic DNA (RAPD) genotyping [16]; AT-typing and eburst analysis [17]; multilocus sequencing typing (MLST), rMLST (ribosomal MLST) and whole-genome MLST [Bacterial Isolate Genome Sequence Database (BIGSdb) [18]. Details of all 103 strains examined and which analyses were performed on each of them are given in Table S1.

Growth curve analysis and swimming, swarming and twitching motility assays were performed as described previously [14]. Preservative susceptibility testing of *P. aeruginosa* was carried out using a microbroth dilution assay essentially as described previously [19] and the minimal inhibitory concentration (MIC) was determined for six widely used industrial agents: chloromethylisothiazolinone (CITMIT; Kathon CG, Dow Europe GmbH, Switzerland); methylisothiazolinone (MIT; neolone M10, Dow Europe GmbH, Switzerland); benzisothiazolinone (BIT; Koralone B-120, Dow Europe GmbH, Switzerland); phenoxyethanol (PHE; Clariant Produkte GmbH, Germany); chlorhexidine (CHX; chlorhexidine digluconate; Sigma-Aldrich Co. Ltd, UK); and benzoic acid (BA; Sigma-Aldrich Co. Ltd, UK) (see Methods S1, Supplementary Material for full details).

A biofilm formation assay was adapted from a previously published protocol [20]. Suspensions of 10^5^ colony-forming units (c.f.u.) ml^−1^ of each strain were prepared in TSB and 100 µl was inoculated into clear 96-well plates, 6 wells per strain (avoiding edge wells). After incubation at 37 °C for 32 h, growth was discarded and the wells were washed three times with sterile H_2_O before staining with 200 µl 0.1 % (w/v) crystal violet solution. The wells were then washed with sterile H_2_O until the washes were clear and left to air-dry overnight. The crystal violet in each well was solubilized with 200 µl 70 % ethanol and the absorbance measured at 570 nm. Three biological replicates were performed for each strain.

## RAPD and AT genotyping of *P. aeruginosa*

RAPD-PCR typing was performed as described previously using primer 272 [5]. RAPD-PCR products were separated using an Agilent 2100 Bioanalyzer and DNA 7500 chips according to the manufacturer’s instructions. Cluster analysis of the RAPD-PCR profiles was performed using the Gel Compar package within Bionumerics 6.6 software (Applied Maths, Gent, Belgium). A Pearson correlation similarity coefficient with an unweighted pair group method with arithmetic mean (UPGMA) dendrogram was used to determine similarity between profiles; strains sharing ≥80 % profile similarity were considered to be the same RAPD type [5].

The Clondiag Array Tube (AT) *P. aeruginosa* genotyping kit (Alere Technologies Gmbh, Jena, Germany) was used to genotype selected industrial/reference strains (Table S2) as described previously [21]. The relatedness of the *P. aeruginosa* strains was determined by comparison of the hexadecimal codes obtained for each strain to a 1464-strong database of clinical and environmental strains (those with complete profiles in the eBURST V3 database [17]) using the default algorithm (http://eburst.mlst.net) [22].

### Draft genome sequencing of *P. aeruginosa*

DNA was extracted from 19 *P*. *aeruginosa* strains (16 industrial strains and 3 reference strains used in industrial testing; Table S3) as described previously [23] (see Method S2, Supplementary Material) and subjected to genome sequencing. Five additional *P. aeruginosa* strains (four clinical, one environmental and one of unknown source) were also subjected to sequencing (Table S3). Genomic library preparation and sequencing were performed at the Oxford Genomics Centre of the Wellcome Trust Centre for Human Genetics. DNA sequencing was achieved using 100 bp paired-end Illumina HiSeq2000 technology. *De novo* assembly of genomes from the paired-end sequence data was performed using SPAdes Genome Assembler v3.5.0. Assembled genomic data in contigs were deposited in the PubMLST database, supported by BIGSdb software [18]. MLST sequencing types (STs) were determined using the PubMLST database. The genomes were deposited in BIGSdb, assigned accession numbers (Table S1; BioProject PRJEB8749) and scanned for the seven *P*. *aeruginosa* MLST loci [24] to identify allele types and STs. The details of other strains in the database sharing the same ST were recorded to determine the origins of the STs.

## Complete sequencing of *P. aeruginosa* RW109

Single molecule real-time (SMRT) sequencing (Pacific Biosciences, PacBio, CA, USA) of *P. aeruginosa* RW109 (ENA accession GCF_900243355.1) was performed by the Centre for Genomic Research, University of Liverpool, essentially as described previously [23]. All bioinformatic analysis of the data was carried out using a virtual machine hosted by the Cloud Infrastructure for Microbial Bioinformatics (CLIMB) consortium [25] and full details of the sequencing and assembly processes are given in the Supplementary Material (Method S3). The assembled genome was analysed as follows: annotation was carried out using Prokka [26]; cluster of orthologous group (COG) was determined as described previously [27]; Kyoto Encyclopedia of Genes and Genomes (KEGG) function was assigned [28] and examined for enrichment in preservative-tolerant strains; phages were located using the PHAge Search Tool Enhanced Release (PHASTER) (v1.0) [29]; and genomic islands were predicted using IslandViewer (v4.0) [30] (see Supplementary Material, Method S4, S5 and S6).

Comparative genome analysis of RW109 was performed against the complete genome sequence FASTA files of the clinical *P. aeruginosa* reference strains UCBPP-PA14 (PA14) (accession no. CP000438) and PAO1 (accession no. AE004091). Each genome was annotated using Prokka and run through the Quality Assessment Tool for Genome Assemblies (QUAST), (v4.5.5) [31], prior to comparison against the RW109 strain. The Circular Genome Viewer (CGView) application and CGView source code [32] were used via the command line to convert Prokka-generated GenBank files into CGView XML files for the generation of an RW109 genome circular map. The coding sequences were coloured according to the assigned COG categories, and the RW109 GIs and prophage sequences were also illustrated on the map.

## *P. aeruginosa* pan-genome analysis

The Roary pipeline (v3.7.0) [33] was used for pan-genome comparisons of the RW109 strain with genome sequences of *P. aeruginosa* strains isolated from clinical, environmental and industrial sources. In total, a reference set of 103 genomes were analysed (Table S1). All the FASTA sequences of the strains were reannotated using Prokka [26] and the resulting GFF files were analysed with Roary [33]. The –e parameter was applied to create a multi-FASTA codon-aware alignment of all the core genes using PRANK [34]. The core-gene multi-FASTA alignment file (MAFFT) was used to build a phylogenetic maximum-likelihood tree based on 1000 bootstrap resampling replicates with FastTree (v2.1.8) [35]. The generalized time-reversible (GTR) model was applied with default settings. The tree was visualized with FigTree (v1.4.3, http://tree.bio.ed.ac.uk/software/figtree/). From the Roary pan-genome analysis, the gene presence and absence output was used to determine the genes specific to the *P. aeruginosa* RW109 strain that were not identified in the 102 *P*. *aeruginosa* genome sequences.

### Prediction of antimicrobial resistance genes

The FASTA sequences of the 103 *P*. *aeruginosa* genomes were analysed using the ABRicate tool (v0.5-dev, https://github.com/tseemann/abricate.git) and antimicrobial resistance genes were predicted by the Comprehensive Antibiotic Resistance Database (CARD) [36]. An ≥80 % cut-off was used for both coverage and identity ABRicate scores. The ABRicate genome summary reports were combined into a single Excel spreadsheet to show the presence and absence of antimicrobial resistance genes. Visualization of the comparative antimicrobial resistance gene content was performed using the Interactive Tree of Life (iTOL) (v3.5.4) [37] by combining the spreadsheet and core-gene phylogenetic tree and producing a heat map.

### Analysis of the RW109 megaplasmid

The CGView Comparison Tool [38] was used to map the *P. aeruginosa* industrial strains, along with the clinical strains PA121617 and PA96, to a circular map of the RW109 plasmid. Prokka-generated GenBank files were used as an input and were converted into CGView XML files for generation of the genome circular comparison map. For evolutionary analysis of the plasmid origin, the *parB* from the RW109 megaplasmid was used to extract homologous sequences by blast from GenBank. A maximum-likelihood phylogenetic tree was generated based on MAFFT alignment of RW109 megaplasmid *parB*, homologous sequences identified in seven industrial and two clinical *P. aeruginosa* strains, and their closest neighbouring *parB* genes from characterized *Pseudomonas* plasmids [39] and other species. The accession numbers of the sequences analysed were as follows: the RW109 megaplasmid 1 (LT969519.1) and plasmids of the same ancestral family, pBM413 (CP016215.1), pSY153-MDR (KY883660.1), p12969-DIM (KU130294.1), pRBL16 (CP015879.1), pJB37 (KY494864.1), pTTS12 (NZ_CP009975.1) and pOZ176 (KC543497.1), and more distantly related plasmids, pTC-F14 (AF325537.2), AMMD p1 (CP000443.1), pAOVO02 (CP000541.1), Rms149 (AJ877225), pRA2 (U88088.2), pWW0 (AJ344068.1) and pUnnamed1 (NZ_CP029091.1; used as the phylogenetic root). The megaplasmid and large plasmid copy numbers were estimated by mapping the short read sequence data to the complete PacBio *P. aeruginosa* RW109 reference sequence using the EDGE bioinformatics platform [40]; the fold coverage of each replicon was compared to that of the main chromosome to derive the copy number estimate.

## Results

### Industrial *P. aeruginosa* tolerate preservatives but are diverse in relation to other phenotypes

**Fig. 1.**
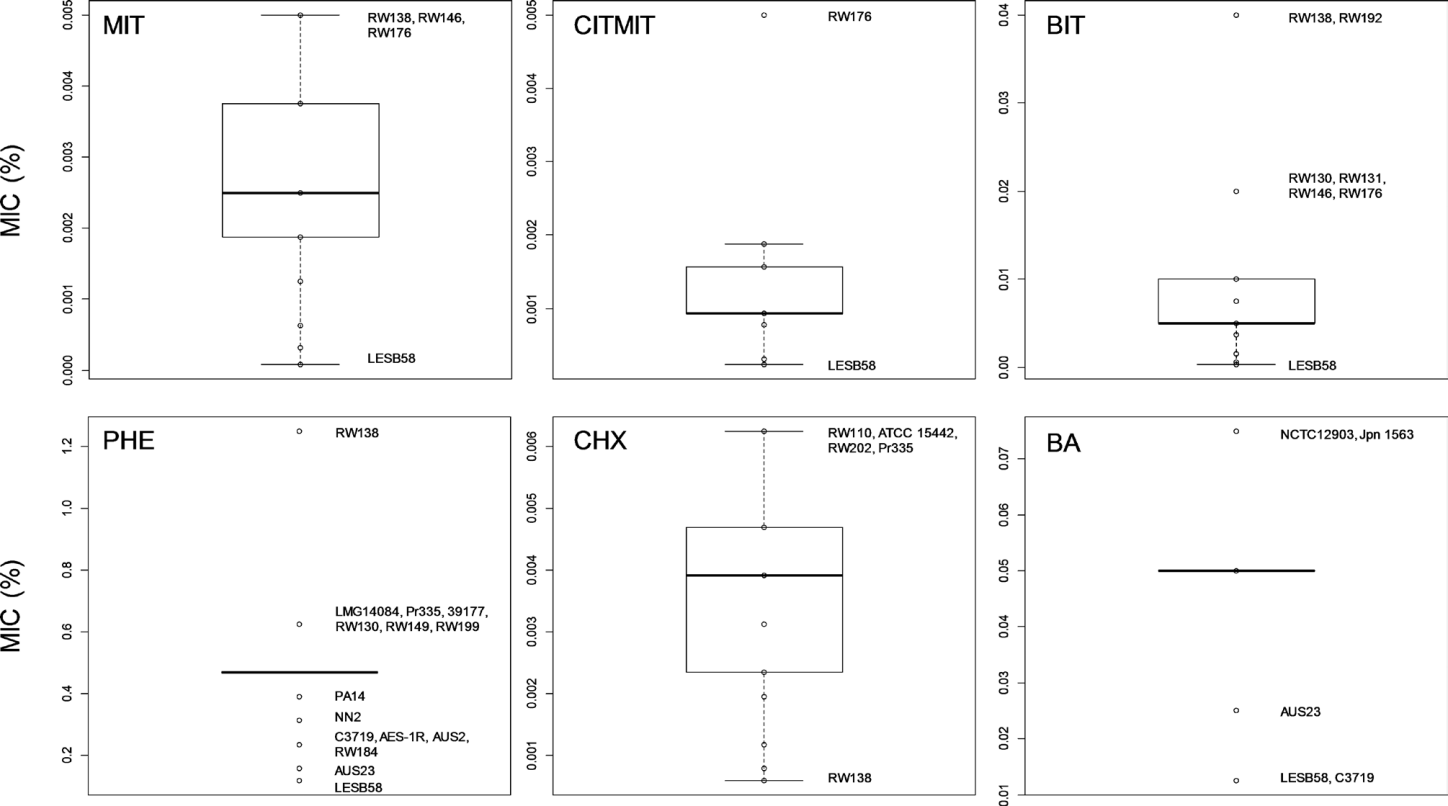
*
P. aeruginosa* demonstrates high tolerance of preservatives commonly used in industry. The minimum inhibitory concentrations (MICs) of preservatives were determined for the sub-panel of 40 *P* *. aeruginosa* strains. MIC data were visualized using boxplots and the median, upper quartile, lower quartile, maximum and minimum values are displayed. Data points are shown as open circles and outliers (minimum and maximum) are labelled with the strain name(s) possessing that MIC. Panels are labelled as follows: MIT, methylisothiazolinone; CITMIT, chloromethylisothiazolinone and methylisothiazolinone blend in a 3 : 1 ratio; BIT, benzisothiazolinone; PHE, phenoxyethanol; CHX, chlorhexidine; and BA, benzoic acid.

A collection of 69 *P*. *aeruginosa* isolates (Table S1) was assembled from industrial products (household cleaners, laundry liquids, personal care cosmetics, metal working fluids and timber care products) that were identified as not meeting hygiene standards during quality control checks of products manufactured between 2001 to 2013; reference stains used in preservative efficacy testing by industry were also included. Low-resolution genotyping (RAPD) was used to differentiate sequential isolates and identify single strains for further analysis (Fig. S1). Representative genetically distinct industrial strains (IND; *n*=15) were assembled into a testing panel of 40 *P*. *aeruginosa* strains from CF (*n*=10), other clinical infections (CLIN; *n*=9), environmental sources (ENV; *n*=5) and unknown (*n*=1) sources. Strains from IPARP [13] were also included (Table S1). Tolerance towards the following widely used preservatives was evaluated: isothiazolinones (methylisothiazolinone, MIT; chloromethylisothiazolinone and methylisothiazolinone blended in a 3 : 1 ratio, CMIT; benzisothiazolinone, BIT), alcohols (phenoxyethanol, PHE), biguanides (chlorhexidine, CHX) and organic acids (benzoic acid, BA) (Fig. 1, Tables S4 and S5).

The industrial strains of *P. aeruginosa* demonstrated high levels of tolerance towards individual preservatives and individual industrial isolates possessed the maximum preservative MIC values for all classes (Fig. 1, Tables S4 and S5). Higher isothiazolinone MIC values (≥0.00375 % MIT, ≥0.001875 % CITMIT and ≥0.02 % BIT) were predominantly linked with industrial strains (Table S4). *P. aeruginosa* strains from industrial sources were found to have significantly higher (*P*<0.05) median preservative MICs than strains from CF for the isothiazolinones (Tables S4 and S5; MIT, 0.0025 compared to 0.001875; CITMIT, 0.0015626 compared to 0.0007815; BIT, 0.01 compared to 0.005; and PHE, 0.4688 compared to 0.2734). Other phenotypes, such as growth rate (Table S6) and motility (Table S7), were variable across the industrial strains. Only one strain, the industrial isolate RW200, demonstrated a true swarming phenotype, indicated by the formation of finger-like projections radiating from the inoculation point (Fig. S2a). In addition, three strains of industrial origin sharing the same genotype (RW130, RW131 and RW146; see below) isolated from a household cleaning product at the same location over several years (2004–2010), displayed the same unusual swimming motility (Fig. S2b).

The ability of the industrial *P. aeruginosa* strains to form biofilms was also investigated [20]. For the 16 industrial product isolates there were no associations between biofilm-forming capability (Fig. S3) and decreased susceptibility to the preservatives MIT, CITMIT, BIT, PHE, CHX or BA (Table 1). Biofilm formation across the 16 industrial, 4 CF, 4 clinical, and 2 environmental strains assessed was variable, but showed concordance with published data in terms of high and low biofilm-forming control strains that were included [14]. No association between strain source or planktonic antimicrobial tolerance and biofilm formation was established (Fig. S3), which was in agreement with what has been observed for *P. aeruginosa* [14] and *Burkholderia cepacia* complex bacteria [41].

**Table 1.** Comparative genome features of the industrial *P. aeruginosa* strain RW109 and the reference strains PA14 and PAO1

**Genome features**	**Industrial strain** **RW109**	**Reference strains**	
		**PA14**	**PAO1**
Genome size (bp)	7756224	6537648	6264404
Replicons	3	1	1
DNA G+C content (%)	65.11	66.29	66.56
Coding sequences	7303	5901	5671
No. hypothetical proteins	2199	2026	1858
rRNA genes	12	12	12
tRNA genes	76	69	73

### Industrial strains are widely spread across the non-clonal, freely recombining *P. aeruginosa* population structure

**Fig. 2.**
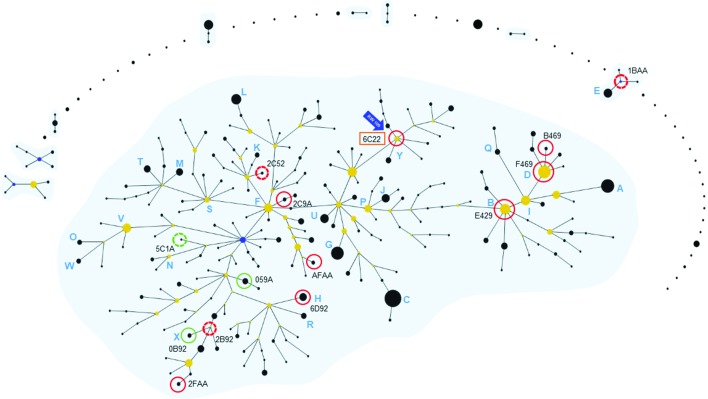
Industrial *P. aeruginosa* strains are widely distributed across the AT genotype inferred population. The clonal complex structures of 1464 strains are shown, including strains from CF, other human infections and environmental sources, as well as 1446 strains analysed by Wiehlmann *et al*. [17]. The industrial and efficacy testing *P. aeruginosa* strains (Table S2) were analysed and placed within the inferred population as follows. Clonal complexes were calculated from the 16-marker AT genotype of the core genome by the eburst algorithm; the major *P. aeruginosa* clonal complex and 8 additional groups have been highlighted with a blue background. AT genotypes are indicated by black, blue and yellow circles, with the diameter of the circle being proportional to the number of isolates with this genotype. Founding AT genotypes are shown in blue, subfounders are in yellow and prevalent clones are marked with light blue letters next to their corresponding AT genotype. The industry-associated *P. aeruginosa* strains are marked with closed (previously reported AT genotypes) or broken (novel AT genotypes) red circles, and their AT genotypes are indicated (Table S2). The position of the industrial strain RW109 is highlighted by the blue arrow and the orange box-enclosed AT genotype.

Multiple genotyping methods of increasing resolution were employed to examine the placement of industrial strains within the non-clonal recombinant *P. aeruginosa* population structure. A total of 19 representative industrial isolates (Fig S1; 16 industrial product isolates and 3 reference strains used in industry) were subjected to Clondiag AT genotyping [17] to enable comparison to the largest dataset of global strains currently available. AT genotypes were obtained for 15 industrial product isolates, revealing 11 different strain types (Table S2). To place these strains in a wider context, eburst [22] was applied to the 1464 isolates [17] comprising the industrial genotypes. The 252 genotypes identified within this collection grouped into 1 large and 8 small clonal complexes, and 44 singletons (Fig. 2). The eburst topology was highly similar to that observed by Cramer and colleagues [42], indicating a diverse spread of strains, with strains from different isolation sources being spread throughout the *P. aeruginosa* population (Fig. 2). In terms of specific AT genotypes, 3 of the 11 (27 %) of industrial genotypes were novel, with the remaining 8 strain types found being previously associated with varied habitats, such as CF, other clinical settings and the natural environment, and previously well-documented *P. aeruginosa* clone types (major clone types Y, D, B and X).

Whole-genome sequencing (WGS) and MLST were used to obtain further information about *P. aeruginosa* population biology [18, 43]. MLST profiles [24] were generated for the industrial strains and represented 10 STs composed of known STs (*n*=7) and novel STs (*n*=3) (Table S3). Comparison to the global MLST database showed that the seven known STs associated with the industrial strains had been encountered in multiple environments, including CF, clinical infections and the environment (Table S3). ST-2730 was associated with three industrial strains (RW130, 131 and 146) isolated from the same household cleaner production facility from 2004 to 2010 (Table S3). This persistent *P. aeruginosa* industrial strain had a high tolerance towards benzoic acid (Fig. 1) and also possessed an atypical swimming motility (Fig. S2). Single industrial *P. aeruginosa* genotypes were also encountered in more than one industrial product type, with ST-111 occurring in two different household cleaning product types (isolates RW192 and RW138), and ST-1342 being encountered within a personal care cosmetic (isolate RW149) and laundry liquid (isolate RW176) product (Table S3).

**Fig. 3.**
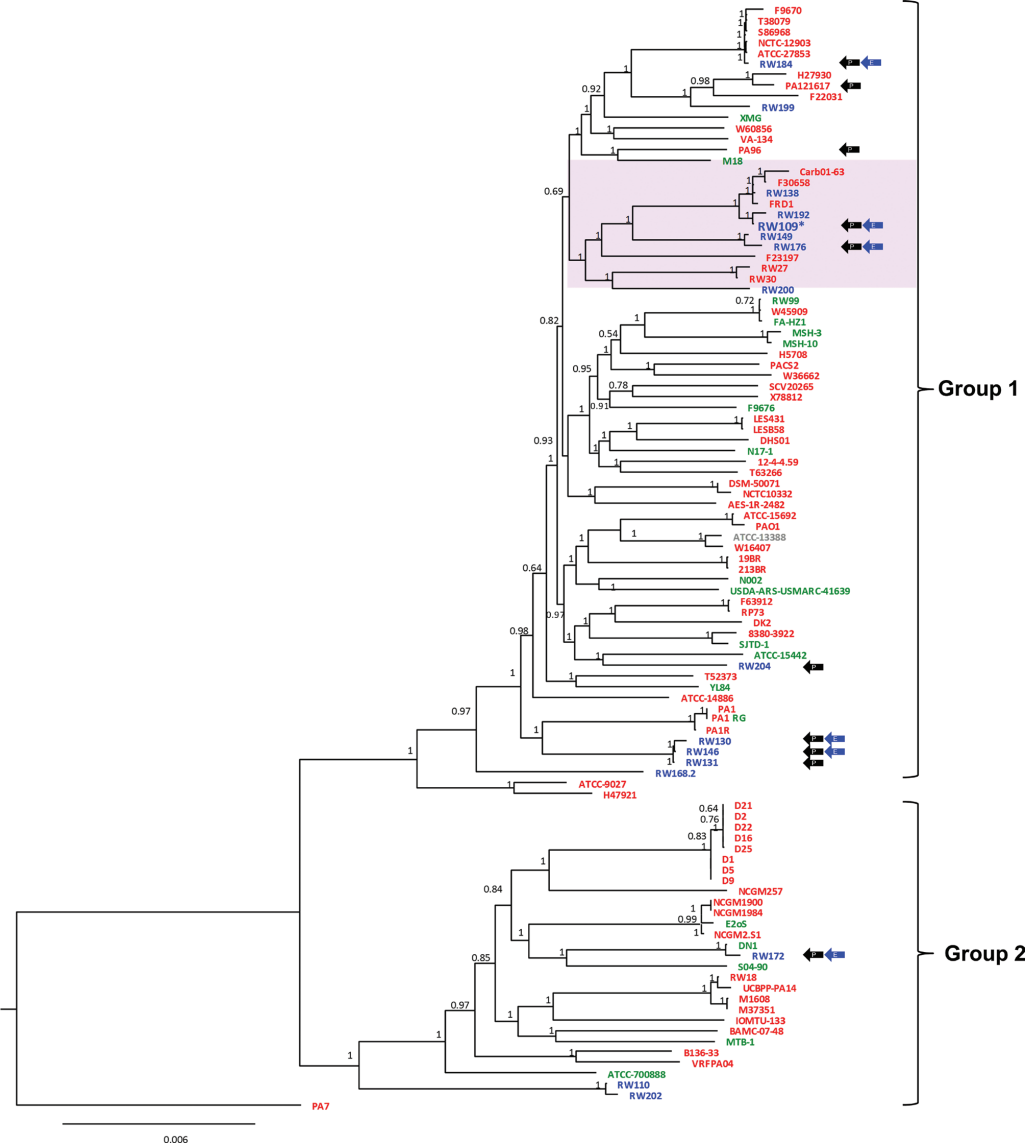
Widespread placement of industrial isolates within the core gene-based population structure of *P. aeruginosa .* A maximum-likelihood phylogenetic tree was generated based on the 4009 core genes identified within the 103 *P*. *aeruginosa* genomes. The strain names are colour coded to indicate their source: clinical (red, *n*=66), environmental (green, *n*=19) or industrial (blue, *n*=16); strain PA1RG is classified as both clinical and environmental and so is coloured red and green, and ATCC-13388 is coloured in grey as it was isolated from an unknown source. Bootstrap values (with 1 being 100 %) and genetic distance (the number of base substitutions per site) are indicated on the nodes and scale bar, respectively. The tree was rooted with the PA7 genome sequence as the most divergent *P. aeruginosa* strain. The two major *P. aeruginosa* groups are labelled (1 and 2), and the highlighted clade (purple square) containing the industrial strain, RW109, with a complete reference genome, is shown. Industrial strains uniquely encoding the BpeEF-OprC efflux pump (blue arrows; E) and the megaplasmid *parB* gene (black arrows; P) are indicated.

High-resolution population biology mapping of the *P. aeruginosa* industrial strains was revealed by core-gene analysis of an expanded genome collection comprising 103 selected sequences (Table S1). A total of 4009 core genes were identified within this dataset, aligned and evaluated phylogenomically (Fig. 3). *P. aeruginosa* split into 2 major groups, with 13 industrial strains in group 1 and 3 in group 2 (Fig. 3). Three industrial strains possessing the same MLST sequence type (ST-111), RW138, RW109 and RW192 (Table S3), were located in a grouping within group 1, and were all differentiated by core gene analysis (Fig. 3). The industrial strains RW149, RW176 and RW200 were also located within this sub-clade, which only comprised industrial and clinical strains (*n*=6 for both; Fig. 3). Strains from different sources were widely dispersed across the core gene population analysis (Fig. 3), corroborating the AT genotyping (Fig. 2). The overall population structure of the two major groups and a PA7 outlier reflects the findings of studies with both smaller (*n*=55) [44] and larger (*n* *=*389 strains) [45] datasets.

### Industrial *P. aeruginosa* strains have large multireplicon genomes

**Fig. 4.**
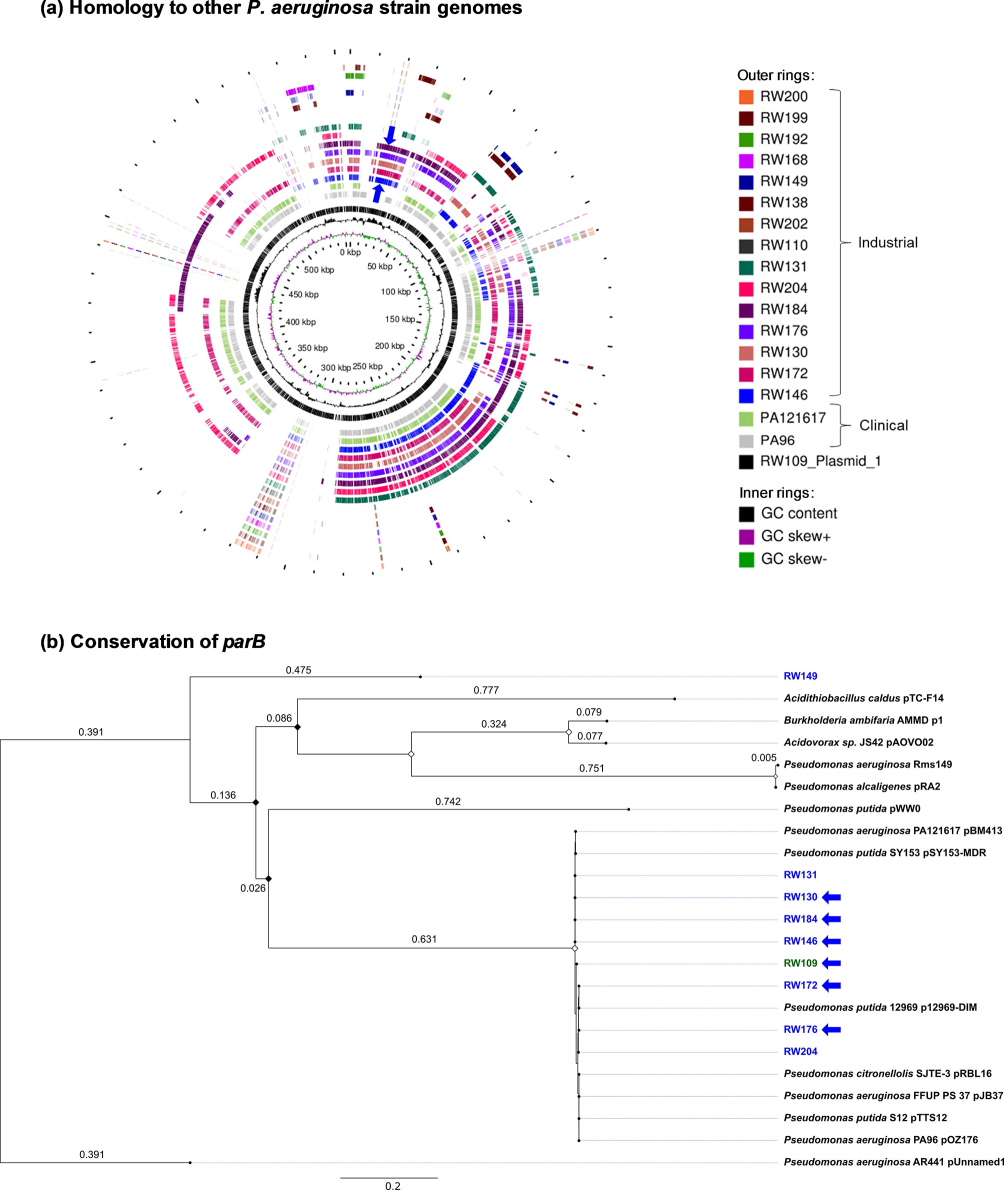
Homology of the RW109 megaplasmid and its conservation among extremely stress-tolerant *Pseudomonas* strains. (a) The CGView Comparison Tool [38] was used to map the *P. aeruginosa* industrial strain sequences, along with the clinical strains PA121617 and PA96, to a circular map of the RW109 megaplasmid. Prokka-generated GenBank files were used as an input and were converted into CGView XML files for generation of the genome circular comparison map. The colour-coded key shows regions of each strain with homology to the megaplasmid, with their source indicated by the parentheses. The blue arrows indicate the approximate location of the conserved BpeEF-OprC efflux pump present in the industrial strains. (b) A maximum-likelihood phylogenetic tree of the RW019 megaplasmid *parB* gene (green text) aligned against homologous sequences from the *P. aeruginosa* industrial strains (blue text) and other related *Pseudomonas* plasmids. Industrial strains uniquely encoding components of the BpeEF-OprC efflux pump are indicated by the blue arrows. Bootstrap values are indicated at the node (diamonds; white nodes ≥75 % and black nodes ≤75 %) and the genetic distance (the number of base substitutions per site) is indicated on the branches and by the scale bar. The tree was rooted with the *parB* gene from an unnamed plasmid in *P. aeruginosa* strain AR441.

To establish a complete reference genome of an industrial *P. aeruginosa* strain, single-molecule real-time sequencing (Pacific Biosciences) was applied to strain RW109, which had the largest genome (Table 1, Fig. S4). This strain had originally been isolated from a product contamination incident (personal care cosmetic product, Europe, 2003; Table S1) and was closely related to five other industrial strains within the same sub-group of group 1 *P*. *aeruginosa* (Fig. 3). In comparison to the well-characterized clinical reference strains, *P. aeruginosa* PA01 and PA14 [46], industrial strain RW109 possessed: (i) a substantially larger genome of 7 756 224 bp; (ii) 1402 more predicted coding sequences than PA14 (the larger of the two well-studied reference strains, PA14 and PA01); and (iii) a multireplicon genome (Fig. 4) comprising a large chromosomal (7 049 347 bp; GC content 65%) replicon, a megaplasmid (555 265 bp; GC content 58 %; plasmid 1) and a large plasmid (151 612 bp; GC content 57 %; plasmid 2) (Table 1, Fig. S4). Other features, such as G+C, rRNA and tRNA content, were similar among the three strains (Table 1). Overall, the highly virulent *P. aeruginosa* PA14 strain genome was 15.7 % smaller, and the older PAO1 reference genome was 19.2 % smaller, than that of the industrial strain RW109

Comparative genome analysis of the *P. aeruginosa* industrial product strains revealed multiple unique traits. Firstly, genes homologous to those encoded by the RW109 megaplasmid were extensively conserved in other genetically distinct industrial strains (Fig. 4a). Comparative genomic analysis of the RW109 megaplasmid demonstrated that seven industrial strains (RW130, RW131, RW146, RW172, RW176, RW184 and RW204) encoded DNA with considerable synteny across >250 kbp of this replicon (Fig. 4a). Two clinical strains, PA121617 and PA96, also encoded DNA that was homologous to the RW109 megaplasmid (Fig. 4a). Collectively, these *P. aeruginosa* strains harbouring DNA homologous to the RW109 megaplasmid were spread widely across the species population structure (seven distinct MLST sequence types; Fig. 3, Table S3). Secondly, evolutionary comparison of the plasmid-specific partitioning gene, *parB* [47], showed that the RW109 megaplasmid was from a single conserved family (Fig. 4b). The RW109 megaplasmid *parB* clustered with the *parB* genes from the same seven industrial strains and two clinical strains with the homologous gene content (Fig. 4a). The close phylogenetic relationship of the *P. aeruginosa* RW109 megaplasmid to characterized plasmids such as pJB37 and pOZ176 [48, 49] indicated that it belonged to the Inc-P2 incompatibility group. By mapping the short-read data to the PacBio complete reference sequence, the megaplasmid copy number was also estimated to be less than 2 (see Supplementary Material, Result S1), which is consistent with the previously reported low copy number associated with Inc-P2 megaplasmids [48, 49]. Overall, from the genomic (Fig. 4a) and phylogenetic (Fig. 4b) conservation, the data suggested that the ancestral backbone of the RW109 megaplasmid had been acquired by different host strains, but was a specific feature of *P. aeruginosa* industrial strains (Fig. 3).

**Fig. 5.**
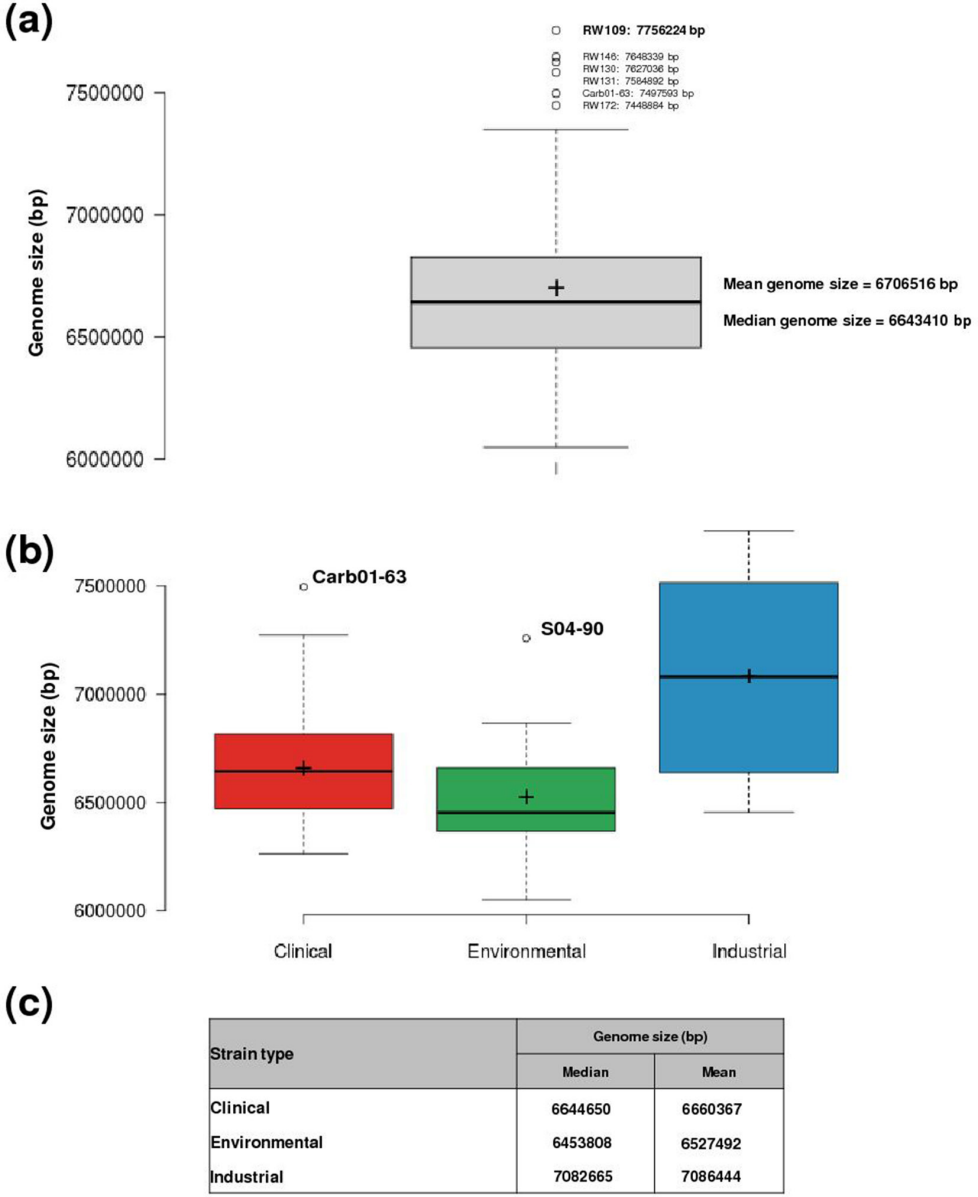
*
P. aeruginosa* strains from industrial product contamination possess considerably larger genomes than those from other sources. The distribution of the genome sizes for the 103 *P*. *aeruginosa* sequences were visualized using boxplots that display the median (black horizontal line), upper quartile, lower quartile, maximum and minimum values. The mean values are also represented with a cross. Outliers are shown as open circles outside of the boxplots, with the strain names and genome sizes indicated. A boxplot to represent the genome size distribution of the 103 sequences combined are shown in (a). The sequences were also divided into three boxplots (b) to indicate the genome sizes of the clinical strains (red, *n*=66), environmental strains (green, *n*=19) and industrial strains (blue, *n*=16). The values for the median and mean genome sizes (bp) for the clinical, environmental and industrial strains are shown in (c).

The third trait shared by all industrial *P. aeruginosa* strains was a larger than average genome size for the species (Fig. 5). The industrial strain genomes were on average 5.7 % (>380 000 bp) larger than *P. aeruginosa* strains from other sources (Fig. 5), despite their genetic heterogeneity at the strain level (Fig. 3). Examination of the genome size distribution for all 103 *P*. *aeruginosa* revealed a mean size of 6 706 516 bp. Five *P. aeruginosa* industrial strains, RW109, RW146, RW130, RW131 and RW172, were outliers possessing the largest genomes in the dataset, with only one other strain, the clinically derived Carb01-63 encoding similar overall DNA content (Fig. 5a). The distribution of genome sizes in relation to strain source (Fig. 5b, c) demonstrated that overall *P. aeruginosa* recovered from industrial settings possessed significantly larger genomes, with a mean size of 7 040 410 bp, compared to clinical strains (mean, 6 660 367 bp; *P*=0.0012) and environmental strains (mean, 6 516 837 bp; *P*=0.0002; two-sample *t*-test assuming unequal variance). In the case of the industrial *P. aeruginosa* strains RW109, RW130, RW131, RW146, RW172, RW176, RW184 and RW204, the presence of the conserved megaplasmid was likely a contributing factor for this increased overall genome size (Fig. 4a, b).

### Industrial *P*. aeruginosa strains have equivalent overall antimicrobial resistance gene content

**Fig. 6.**
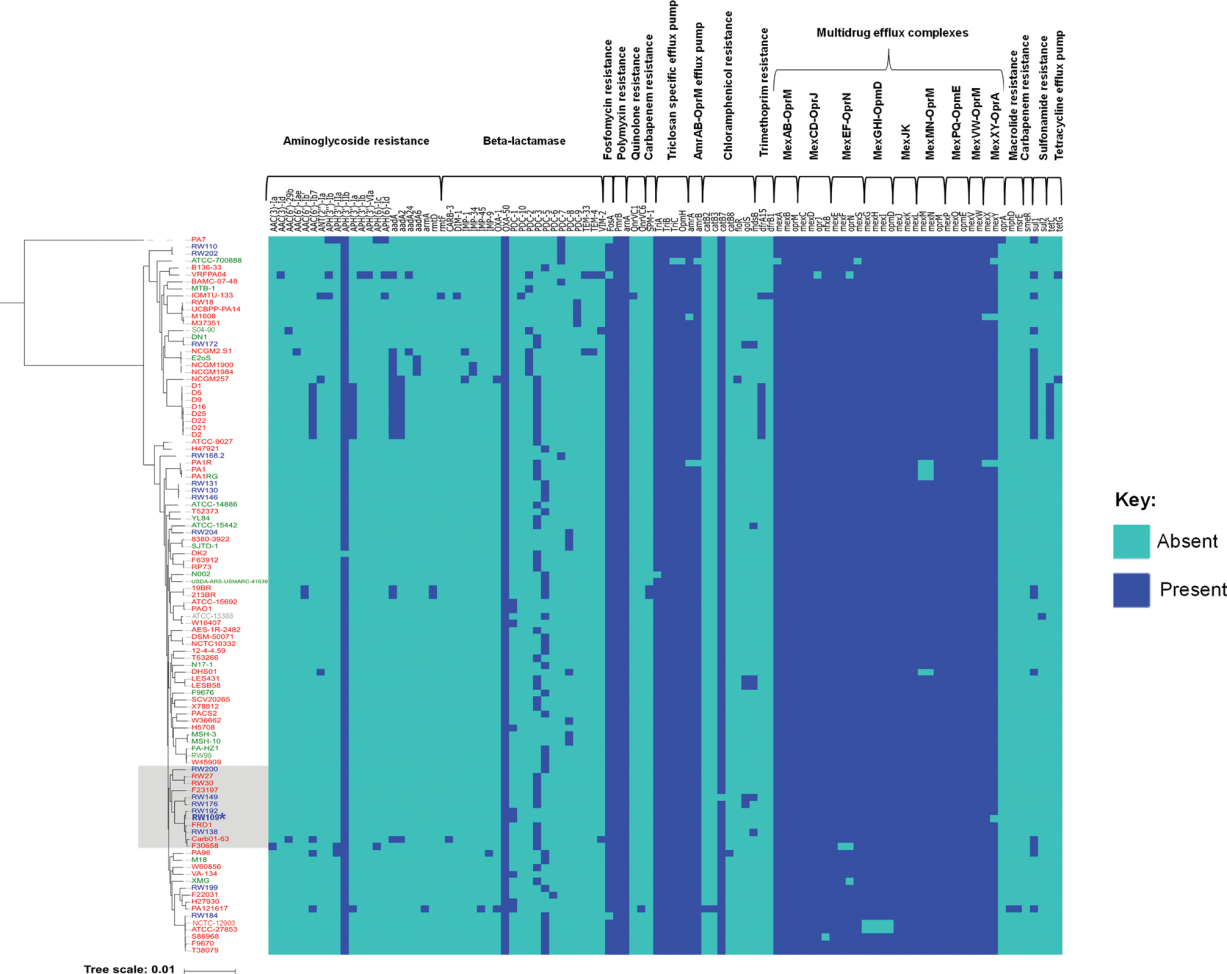
Predicted antimicrobial resistance gene content of the 103 *P* *. aeruginiosa* genomes. Using core-gene phylogenomic analysis to place each *P. aeruginosa* genome in evolutionary context, a presence/absence heat map was used to visualize the CARD-predicted antimicrobial resistance gene content. *P. aeruginosa* genome sequences of clinical (red), environmental (green) and industrial (blue) strains are indicated. The CARD antimicrobial genes were grouped via their predicted resistance functions (top of the heat map), with light blue showing the absence and dark blue indicating the presence of the antimicrobial resistance gene. The clade containing the industrial reference strain, RW109, for which a complete genome was determined, is highlighted (purple box).

Antimicrobial resistance (AMR) genes were predicted from 103 *P*. *aeruginosa* genomes using the CARD [50]. Visualization of the predicted AMR genes demonstrated that industrial strains encoded a similar range of antibiotic resistance genes to *P. aeruginosa* strains from other environments (Fig. 6). The mean number of CARD resistance genes was determined as 43 for CF and clinical strains, 41 for environmental strains and 41 for industrial strains. All 103 genome sequences had the polymyxin resistance genes *pmrB* and *arnA* and the aminoglycoside resistance gene *OXA-50*. Within the industrial genome sequences, RW109 and RW192 were the only two with the beta-lactamase PDC-1 gene; this gene was also found within seven clinical strains (Fig. 4; ATCC-15692, H27930, H5708, PA121617, PAO1, VA-134 and W16407). CARD did not identify any antimicrobial resistance genes associated with the quinolone, carbapenem, trimethoprim, macrolide and tetracycline efflux resistance functional groups. The majority of the genes that encode the Mex-type efflux pump systems were found in all *P. aeruginosa* industrial strains; exceptions were *oprA* (absent from all industrial strain genomes) and the *mexY* gene, which was specifically absent in RW109.

Pan-genome and presence/absence analysis was performed with the 103 *P* *. aeruginosa* genome dataset to determine genes unique to RW109. A total of 329 genes were found to be specific to the industrial strain, with 202 encoded on the main chromosome, 105 on megaplasmid 1 and 22 on plasmid 2. Over half (51%) of the RW109 unique genes were identified as poorly characterized by COG annotation assessment. No CARD-predicted antimicrobial resistance genes were found within the unique subset of RW109 genes. However, five genes associated with a BpeEF-OprC type efflux pump [51] identified by KEGG analysis [module M00698, module completeness ratio (MCR) of 100 % and *Q*-value of 0) were encoded by the RW109 megaplasmid (genes RW109_00010 through RW109_00014). he industrial strains RW130, RW146, RW172, RW176 and RW184, all of which likely possessed a megaplasmid from the same family of as that of RW109 (Fig. 4), also encoded the same KEGG-predicted efflux pump genes (MCR of 100 % and a *Q*-value of 0). Genes homologous to the BpeEF-OprC type efflux pump were not identified in any of the remaining 97 *P* *. aeruginosa* genome sequences. The industrial strains possessing the BpeEF-OprC efflux pump genes were genetically distinct and spread across both major groups of the *P. aeruginosa* population structure (Fig. 3).

KEGG functional module pathway analysis was carried out on four isolates with the highest MICs and four with the lowest MICs for the preservatives BIT, MIT, CITMIT, PHE and CHX (see Fig. 1; Tables S4 and S5). Overall, there were minimal differences in the numbers of complete modules assigned to the categories between the isolates with high and low preservative MICs (Figs S5 and S6). Significant differences were associated with a limited number of functional module pathways, including drug resistance, central carbohydrate metabolism, bacterial secretion system and two-component regulatory system categories (see Supplementary Results; Figs S5 and S6).

## Discussion

Researchers commonly portray *P. aeruginosa* as ubiquitous, linking this descriptor to its diverse phenotypic and genotypic traits, and multiple isolation sources. As an opportunistic pathogen, *P. aeruginosa* has been the subject of extensive investigation, but outside of clinical settings it has received much less attention despite its problematic nature when encountered. With the number of consumer products including preservatives increasing [52], and the production volume of biocidal compounds being several orders of magnitude higher than that of antibiotics [53], industrial manufacturing environments represent a key niche for stress-tolerant organisms such as *P. aeruginosa *. However, in contrast to infection, where *P. aeruginosa* isolates are frequently genotyped and archived to enable global epidemiological analysis, no systematic collections of *P. aeruginosa* isolates from industrial contamination have been assembled. By establishing a unique collection of *P. aeruginosa* isolates from industrial sources spanning a decade, different global locations and multiple product types, we have shown that they possess multireplicon genomes encoding 300 kb of extra DNA compared to strains from other sources.

Preserved industrial products represent an environment where antimicrobial compounds may exist within a concentration gradient ranging from lethal to sub-lethal concentrations [52], and such conditions place huge selective pressure on potential contaminating micro-organisms such as *P. aeruginosa .* Understanding the fitness advantage that *P. aeruginosa* gains from retaining genomic and megaplasmid content for survival within industrial environments is vital to develop improved preservation strategies and formulations. The International *Pseudomonas aeruginosa* Consortium is sequencing over 1000 genomes to study genome evolution, antibiotic resistance and virulence genes [45]. The initial findings from this project examining the core genomic content of 389 strains identified 3 major phylogenetic groups: group 1, which contained well-studied reference strains such as PAO1 [54] and LESB58 [55]; group 2, where the virulent reference strain PA14 [56] resided; and group 3, containing isolate PA7 [57] as an outlier. Our core gene analysis of a representative set of 103 *P* *. aeruginosa* genomes mirrored this analysis, and moreover has shed light on the widespread dispersal of industrial strains within both major phylogenetic groups (Fig. 3).

The consensus population structure of *P. aeruginosa* is non-clonal epidemic, composed of freely recombining isolates without habitat selection, but with evidence of globally distributed major clone types such as clone C and PA14 [3]. Confounding this model are potential niche specialists in clinical and environmental settings [3, 58–60]. The largest *P. aeruginosa* population structure analysis to date used AT genotyping to examine 2192 isolates from 1448 independent habitats and included 8 isolates from oil-contaminated environments, but did not evaluate representative strains from contaminated industrial products [17]. We found that these industrial *P. aeruginosa* strains were diverse and a mixture of novel and previously encountered AT genotypes (Fig. 2). A recent small-scale study of animal infection as an under-reported niche of *P. aeruginosa* found the same diversity of genotypes within carbapenem-resistant strains [61]. The industrial AT genotypes were spread widely throughout the topology of the eburst analysis (Fig. 2), corroborating the non-clonal epidemic hypothesis of *P. aeruginosa* population structure [42]. Furthermore, investigating the antimicrobial resistance gene content did not provide an obvious genetic link to the increased preservative tolerance of the industrial strains (Fig. 1), as strains of different origin shared similar numbers of core genome antimicrobial resistance determinants (Fig. 6). Sharing of resistance capabilities and a lack of clonal specialization in relation to antimicrobial phenotypes has previously been reported for *P. aeruginosa* strains from different environments [62].

Despite this population mixing and multiple ubiquitous traits being shared among *P. aeruginosa* strains, our genomic analysis of industrial strains demonstrated specialization in the direction of greater genomic capacity and multiple replicon genomes. It is interesting that *Burkholderia *, well known for their multireplicon genome structure, are also highly stress-tolerant and intrinsically antimicrobial-resistant [63]. A key feature of the reference industrial genome for *P. aeruginosa* strain RW109 defined herein was the presence of a 555 kbp megaplasmid (Fig. 4). Megaplasmids are well known to encode a wide range of accessory fitness traits that contribute to specialist ecological lifestyles [47]. The RW109 megaplasmid origin was conserved within seven other industrial strains and its gene content was homologous to megaplasmids within *Pseudomonas* species recovered from a wide variety of stressful environments (Fig. 4a, b). These *Pseudomonas* megaplasmids (all >370 kbp) have each been characterized individually for their fitness-contributing roles in clinical multidrug resistance for pSY153-MDR, pBM413, p12969-DIM, pOZ176 [64] and pJB37 [48]; mediating biodegradation processes such as oestrogen and aromatic hydrocarbon breakdown in wastewater treatment (pRBL16) [65]; and solvent tolerance and the production of aromatic hydrocarbons (pTTS12) [66]. The *Pseudomonas putida* megaplasmid, pTTS12 (583 kbp) is the largest characterized for the genus to date [66], and the RW109 megaplasmid (555 kbp) uniquely identified in this study is the largest associated with a strain of *P. aeruginosa *. Recent analysis of *P. aeruginosa* isolates recovered from patients at a hospital in Thailand also showed that the same closely related family of megaplasmids encode multidrug resistance elements, are highly recombinogenic and mediate extensive gene transfer, and have likely been carrying resistance genes dating back to the late 1970s [67].

Specific characterization of this unique megaplasmid family in relation to the wider fitness they bring to *Pseudomonas *, as well as their role in preservative tolerance within industrial isolates, is worthy of further work. Our initial analysis has demonstrated no clear association of megaplasmid with *P. aeruginosa* phenotypes such as growth rate (Table S6), motility (Table S7) or biofilm formation (Fig. S3), but overall the industrial strains harbouring the plasmid showed elevated levels of preservative tolerance (Fig. 1). The KEGG functional module content was also broadly the same for the comparison of the limited numbers of well-characterized preservative-tolerant and -sensitive *P. aeruginosa* strains we were able to compare (Figs S5 and S6). It was interesting that the bacterial secretion category was enriched in isothiazolinone-tolerant *P. aeruginosa* strains (Fig. S5), and that the BpeEF-OprC type efflux pump was an underpinning pathway in this functional content difference (Supplementary Material, Result S2). Systematic comparison of large numbers of *P. aeruginosa* strains and functional analysis of the implicated gene pathways will be required to fully validate these initial correlations. While projects such as the 1000 genomes study [45] are delivering extensive sequence information, more data for poorly studied phenotypes such as preservative tolerance are required to perform high-resolution comparative analysis of *P. aeruginosa .*

## Conclusions

*P. aeruginosa* is rightly described as an opportunistic pathogen, but the data presented here point towards the fact that it is actually a highly opportunistic organism, possessing genomic flexibility that enables it to take advantage of new niches wherever it can find them. The current study has uniquely characterized a collection of *P. aeruginosa* strains of industrial origin that are impacting on global product manufacture and has increased our understanding of this important bacterial species. *P. aeruginosa* is a common contaminant in xenobiotic settings and we identified problematic strains with high preservative tolerance that were capable of causing recurrent industrial incidents. These findings suggested that industrial strains possessed elevated fitness levels and present a case for strain typing and tracking within manufacturing environments as part of quality control. Further investigation into preservative tolerance will inform preservative system development and evaluation, but will require appropriate model strains. The prototypic strains PAO1 and PA14 [46] had significantly smaller genomes and lower preservative tolerance than the industrial strains. We focused our attention on *P. aeruginosa* RW109 and defined a complete reference genome for this industrial strain, discovering that it possessed one of the largest genomes and a megaplasmid associated with the species. Whilst there may not be specialized clones within industry, understanding the potential fitness advantage of the extra genomic content and megaplasmids shared by *P. aeruginosa* industrial strains is a key future challenge.

## Data bibliography

1. Weiser RW, Bull MJ, Jolley KA, Maiden MCJ and Mahenthiralingam E. European Nucleotide Archive, PRJEB8749 (2015).

## Supplementary Data

Supplementary File 1
