# Supplementary material for: Not all Pseudomonas aeruginosa are equal: strains from industrial sources possess uniquely large multireplicon genomes
Source: Microb Genom. 2019 Jun 6;5(7):e000276. doi: 10.1099/mgen.0.000276 (PMC6700666; doi:10.1099/mgen.0.000276)
Supplement: Supplementary File 1 [file mgen-5-276-s001.pdf]

Supplementary Material File

Not all *Pseudomonas aeruginosa* are equal: strains from industrial sources possess uniquely large multireplicon genomes

Running title: Industrial *P. aeruginosa* genomics

Author names: Rebecca Weiser,[a*] Angharad E. Green,[a, c*] Matthew J. Bull,[a] Edward Cunningham-Oakes,[a] Keith A. Jolley,[b] Martin C.J. Maiden,[b] Amanda J. Hall,[c] Craig Winstanley,[c] Andrew J. Weightman,[a] Denise Donoghue,[d] Alejandro Amezquita,[e] Thomas R. Connor,[a] and Eshwar Mahenthiralingam[a#]

*** R.W and A.E.G contributed equally to this work**

Affiliation:

[a] Microbiomes, Microbes and Informatics Group, Organisms and Environment Division, Cardiff School of Biosciences, Cardiff University, Cardiff, Wales, UK.

[b] Department of Zoology, The Tinbergen Building, University of Oxford, Oxford, UK.

[c] University of Liverpool, Institute of Infection and Global Health, Liverpool, UK.

[d] Unilever Research and Development, Port Sunlight, Wirral, UK.

[e] Unilever Research and Development, Safety & Environmental Assurance Centre, Colworth House, Sharnbrook, Bedford, UK.

[#]Corresponding author: Eshwar Mahenthiralingam, MahenthiralingamE@cardiff.ac.uk (ORCID: 0000-0001-9014-3790)

Key words: *Pseudomonas aeruginosa*; Industry microbiology; Contamination; Phylogenomics; Megaplasmids.

Table of Contents

Content	Title	Page
**Methods**		
Method S1	Preservative susceptibility testing	3
Method S2	Extraction of DNA from *P. aeruginosa*	3
Method S3	Complete genome sequencing and annotation of *P. aeruginosa* RW109	3
Method S4	Assigning functional groups to the *P. aeruginosa* RW109 genome sequence	4
Method S5	Kyoto Encyclopedia of Genes and Genomes (KEGG) functional module assignment	5
Method S6	KEGG enrichment analyses in relation to preservative tolerance	5
**Results**		
Result S1	The *P. aeruginosa* RW109 megaplasmid copy number.	6
Result S2	KEGG functional module enrichment and *P. aeruginosa* preservative tolerance	6
**References**		7-9
**Tables**		
Table S1	Collection of industrial and other *P. aeruginosa* strains assembled and analysed in this study	10-16
Table S2	ArrayTube (AT) genotypes of industrial and reference testing *P. aeruginosa* strains	17
Table S3	MLST allele and Sequence Type (ST) designations for industrial and reference testing *P. aeruginosa* strains	18
Table S4	*P. aeruginosa* tolerance of the isothiazolinone preservatives MIT, CITMIT and BIT	19-20
Table S5	*P. aeruginosa* tolerance of the preservatives phenoxyethanol, chlorhexidine and benzoic acid	21-22
Table S6	Growth parameters of *P. aeruginosa* strains in liquid culture after 24 hours growth	23
Table S7	Swimming, swarming and twitching motilities of *P. aeruginosa*	24
**Figures**		
Figure S1	Clustered RAPD-PCR profiles of 69 industrial *P. aeruginosa* isolates	25
Figure S2	Swimming, swarming and twitching motilities of the *P. aeruginosa* strains.	26
Figure S3	Biofilm formation by *P. aeruginosa* panel and industrial strains	27
Figure S4	The complete multireplicon genome of industrial *P. aeruginosa* strain RW109	28
Figure S5	KEGG functional module enrichment analysis in relation to *P. aeruginosa* tolerance of BIT, MIT and CITMIT	29
Figure S6	KEGG functional module enrichment analysis in relation to *P. aeruginosa* tolerance of PHE and CHX	30

Supplementary Methods

Method S1. Preservative susceptibility testing. Broth MIC determination for individual preservatives was performed essentially as described [1]. Aqueous stock solutions of preservatives were diluted (serial doubling dilutions) in TSB (Oxoid Ltd, Basingstoke, UK) to produce a range of concentrations: 0 – 0.01% Chloromethylisothiazolinone (CITMIT; Kathon CG, Dow Europe GmbH, Switzerland); 0 – 0.01% Methylisothiazolinone (MIT; Neolone M10, Dow Europe GmbH, Switzerland); 0 – 0.08% Benzisothiazolinone (BIT; Koralone B-120, Dow Europe GmbH, Switzerland); 0 – 2.5% phenoxyethanol (PHE; Clariant Produkte GmbH, Germany); 0 – 0.1% Chlorhexidine (CHX; chlorhexidine digluconate; Sigma-Aldrich Co. Ltd., Poole, UK); and 0 – 0.4% benzoic acid (BA; Sigma-Aldrich Co. Ltd., Poole, UK). Dilutions of BA were prepared in TSB at pH 5. Approximately 10^5 cfu of *P. aeruginosa* isolates were inoculated into 96-well microplates containing 200 µl of preservative concentration per well, followed by shaking incubation (150 rpm) for 24 hours at 37°C. The optical density of each well at 600 nm was recorded using a microplate reader (Tecan Infinite® 200 PRO; Tecan UK Ltd., Reading, UK). The MIC was taken as the concentration of preservative at which there was an 80% reduction in OD from TSB growth control wells. Two biological replicates, each with two technical replicates were performed. The MIC data were analysed as discreet data using non-parametric statistical methods. The median of the four replicates was recorded to obtain final MIC values. Boxplots summarised the distribution of the MIC data for each preservative. Comparisons between strain groups from different isolation sources were performed using a Kruskal-Wallis test and post-hoc Wilcoxon tests with Benjamini-Hochberg correction. The group medians were deemed statistically significantly different at the p=0.05 level.

Method S2. DNA extraction. Extraction of *P. aeruginosa* genomic DNA from fresh overnight cultures was achieved as described [2] using the Maxwell® 16 instrument and the Maxwell® 16 Tissue DNA purification kit (Promega, Southampton, UK) according to the manufacturer's instructions. DNA was RNase A treated before long-term storage at - 20°C. DNA quality and quantity were assessed using a NanoDrop Spectrophotometer (Thermofisher Scientific, Massachusetts, USA) and a Qubit™ fluorometer with the Qubit™ dsDNA BR assay kit (Invitrogen, Massachusetts, USA), respectively.

Method S3. Complete genome sequencing and annotation of *P.* aeruginosa RW109. Two Single Molecule, Real-Time (SMRT) cells with P6/C4 chemistry on a Pacific Biosciences (PacBio, California, USA) RSII, were used to generate the raw sequence data. Subsequent assembly and bioinformatic analysis was carried out using a virtual machine hosted by the Cloud Infrastructure for Microbial

Bioinformatics (CLIMB) consortium [3]. The following protocol was used to create FASTQ DNA sequence files from the raw data PacBio files. From each sequencing run, three bax.h5 files and one bas.h5 file (a pointer file to the three bax.h5 files) resulted from each SMRT cell. The bax.h5 files contained the base call information from the sequencing run, and both sets from the two SMRT cells were converted into two separate binary format (bam) files using the bax2bam tool (v0.0.8, PacBio). The two resulting bam files were merged and a single FASTQ was extracted using the BamTools toolkit (v2.4.0, https://github.com/pezmaster31/bamtools). Assembly of the FASTQ sequence data was carried out as follows. The contigs were created from FASTQ files using the Canu Assembler (v1.3) [4]. Assembled contigs were checked for overlapping ends and trimmed where necessary using Circulator (v1.2.1) [5]. The resulting assembly was polished using the Genomic Consensus Package (v2.1.0, PacBio) The FASTA sequence file of the RW109 genome was run though the Quality Assessment Tool for Genome Assemblies (QUAST), (v4.5.5) [6]. The complete RW109 genome is available at https://www.ebi.ac.uk/ena/data/view/GCA_900243355.1.

Method S4. Assigning functional groups to the *P. aeruginosa* RW109 genome sequence. The COG functional annotation [7] of the RW109 Prokka predicted coding sequences [8] (CDS; specifically translated amino acid sequences) was carried out with the command line EggNOGmapper downloaded from the evolutionary genealogy of genes: Non-supervised Orthologous Groups (EggNOG) database (v4.5.1) . Functional orthologs were assigned using the EggNOG HMMER3 [9, 10] based homology search to the optimised bacterial database and the COG categories and accession numbers were extracted. COG categories were divided into three well characterised functional classes' information storage and processing, cellular processes and signalling and metabolism. A poorly characterised functional class was also used where the COG category was unknown.

RW109 genomic islands (GIs) were predicted using Islandviewer (v4.0) [11] with the Prokka generated GeneBank file with default settings applied. The results from IslandViewer prediction methods SIGI-HMM and IslandPath-DIMOB was used to identify the total number of GIs within the RW109 whole genome sequence. Prophage sequences within the RW109 genome were predicted using PHAge Search Tool Enhanced Release (PHASTER) (v1.0) [12]. Comparisons against the PHASTER databases and feature identifications were carried out with the Prokka generated GeneBank file. Phage sequence regions were given a PHASTER score and were identified as being complete if the score was > 90, questionable with a score of 70-90 and incomplete with a score < 70. The ABRicate tool (v0.5-dev, https://github.com/tseemann/abricate.git) via the command line was used to screen the Prokka annotated nucleotide sequence of RW109 to identify antimicrobial and virulence genes using the

Comprehensive Antibiotic Resistance Database (CARD) 2013) [13]. A ≥80% cut off was used for both coverage and identity ABRicate scores.

Method S5. Kyoto Encyclopedia of Genes and Genomes (KEGG) functional module assignment. KEGG Orthology (KO) terms were assigned to the RW109 translated amino acid sequences from Prokka predicted CDS, using the KEGG Automatic Annotation Server (KAAS) [14] through the NCBI BLAST single-directional best hit search method, against the prokaryotes organism list. The Metabolic and Physiological Potential Evaluator (MAPLE) tool (v2.1.0) [15], was subsequently used to map the groups of KO-assigned CDS to KEGG defined modules. These modules are a collection of functional units linked to specific metabolic abilities and phenotypic features, and identified with M numbers. KEGG modules are grouped into pathway modules, structural complexes, functional sets and signature modules [15]. The percentage of a module's completeness was determined by calculating the module completion ratio (MCR), which evaluated how many KO components of the module were present. If all KO assigned CDS within each module were present, the MCR was equal to 100%, according to a Boolean algebra-like equation [15]. For each MCR, a Q-value was also calculated which indicated the significance of the module completion. The MAPLE tool inferred that reaction modules with Q-values of less than 0.5 were biologically feasible even if the MCR was less than 100% [15].

Method S6. KEGG enrichment analyses in relation to preservative tolerance. For each preservative (BIT, MIT, CITMIT, PHE and CHX), 4 isolates with the highest and 4 with the lowest MIC were selected for comparative analysis of KEGG functional modules as follows. After repeat annotation with Prokka [8], KEGG Orthology (KO) terms were assigned to the CDS of the translated amino acid sequences for each strain, using the KEGG Automatic Annotation Server [14]. The Metabolic and Physiological Potential Evaluator (MAPLE) tool (v2.1.0) [15] was subsequently used to map the groups of KO-assigned CDS to KEGG defined modules (modules constituted collections of functional units linked to specific metabolic abilities and known phenotypic features). The KEGG modules were grouped by category and the number of complete modules for each category were compared. A Two-way ANNOVA with Sidak's multiple comparisons test was used to look for significantly different KEGG module numbers between the strains with high versus low preservative MIC.

Supplementary Results

Result S1. The *P. aeruginosa* RW109 megaplasmid copy number. Mapping of short reads to the complete RW109 sequence using the EDGE software [16] derived fold coverage metrics of 36 ± 9.9,

67 ± 25 and 100 ± 30 (standard deviation) for the main chromosome, megaplasmid and large plasmid respectively. The copy number of the megaplasmid was estimated to be less than 2 since the sequence coverage was 1.8-fold greater than that of the main chromosome. The Inc-P2 plasmids that were phylogenetically closely related to the *P. aeruginosa* RW109 megaplasmid (pJB37 and p0Z176; Figure 4) are also predicted to be low copy number [17, 18]. The RW109 large plasmid (Figure S4) was also likely a low copy number given that plasmid sequence coverage was 2.7-fold greater than that for the main chromosome.

Result S2. KEGG functional module enrichment and *P. aeruginosa* preservative tolerance. KEGG functional module pathway analysis was carried out on the four *P. aeruginosa* isolates with the highest MICs and four with the lowest MICs for the preservatives BIT, MIT, CITMIT, PHE and CHX (Figure 1; Table S4 and S5). The modules were grouped by category and the number of complete modules for each category were compared for the isolates with high MIC versus the isolates with low MIC for each preservative. Overall, there were minimal differences in the numbers of complete modules assigned to the categories between *P. aeruginosa* with high and low preservative MICs, although significant differences were identified for a small number of categories (Figure S5 and S6). Isolates with higher BIT and MIT MICs had a significantly higher number of modules assigned to the drug resistance category (Figure S5). Interestingly this was due to the addition of the complete multidrug resistance efflux pump BpeEF-OprC module in the isolates RW176 and RW146, which had high BIT and MIT MICs. *P. aeruginosa* isolates with low MICs for BIT and MIT were found to have a significantly higher number of complete modules assigned to central carbohydrate metabolism when compared to those with higher MICs for these preservatives (Figure S5). Isolates with high CITMIT (Figure S5) and PHE (Figure S6) MICs had significantly more modules categorised as two component regulatory systems. A higher number of modules were identified in the bacterial secretion system category for isolates with high BIT and MIT MICs (Figure S5). Isolates with high CHX MICs were found to have significantly higher modules identified in the central carbohydrate metabolism category (Figure S6). Detailed functional analysis of the implicated pathways and screening of additional *P. aeruginosa* strains is required to expand on these interesting preliminary findings.

Supplementary References

1. **Rushton L, Sass A, Baldwin A, Dowson CG, Donoghue D et al.** Key role for efflux in the preservative susceptibility and adaptive resistance of *Burkholderia cepacia* complex bacteria. *Antimicrob Agents Chemother* 2013;57(7):2972-2980.
2. **Song L, Jenner M, Masschelein J, Jones C, Bull MJ et al.** Discovery and Biosynthesis of Gladiolin: A *Burkholderia gladioli* Antibiotic with Promising Activity against Mycobacterium tuberculosis. *J Am Chem Soc* 2017;139(23):7974-7981.
3. **Connor TR, Loman NJ, Thompson S, Smith A, Southgate J et al.** CLIMB (the Cloud Infrastructure for Microbial Bioinformatics): an online resource for the medical microbiology community. *Microb Genom* 2016;2(9):e000086.
4. **Koren S, Walenz BP, Berlin K, Miller JR, Bergman NH et al.** Canu: scalable and accurate long-read assembly via adaptive k-mer weighting and repeat separation. *bioRxiv* 2017:071282.
5. **Hunt M, De Silva N, Otto TD, Parkhill J, Keane JA et al.** Circlator: automated circularization of genome assemblies using long sequencing reads. *Genome biology* 2015;16(1):294.
6. **Gurevich A, Saveliev V, Vyahhi N, Tesler G**. QUAST: quality assessment tool for genome assemblies. *Bioinformatics* 2013;29(8):1072-1075.
7. **Tatusov RL, Galperin MY, Natale DA, Koonin EV**. The COG database: a tool for genome-scale analysis of protein functions and evolution. *Nucleic Acids Res* 2000;28(1):33-36.
8. **Seemann T**. Prokka: rapid prokaryotic genome annotation. *Bioinformatics* 2014;30(14):2068-2069.
9. **Huerta-Cepas J, Forslund K, Szklarczyk D, Jensen LJ, von Mering C et al.** Fast genome-wide functional annotation through orthology assignment by eggNOG-mapper. *bioRxiv* 2016:076331.
10. **Huerta-Cepas J, Szklarczyk D, Forslund K, Cook H, Heller D et al.** eggNOG 4.5: a hierarchical orthology framework with improved functional annotations for eukaryotic, prokaryotic and viral sequences. *Nucleic Acids Res* 2016;44(D1):D286-D293.
11. **Bertelli C, Laird MR, Williams KP, Lau BY, Hoad G et al.** IslandViewer 4: expanded prediction of genomic islands for larger-scale datasets. *Nucleic Acids Res* 2017:doi: 10.1093/nar/gkx1343.
12. **Arndt D, Grant JR, Marcu A, Sajed T, Pon A et al.** PHASTER: a better, faster version of the PHAST phage search tool. *Nucleic Acids Res* 2016;44(W1):W16-W21.
13. **McArthur AG, Waglechner N, Nizam F, Yan A, Azad MA et al.** The Comprehensive Antibiotic Resistance Database. *Antimicrob Agents Chemother* 2013;57(7):3348-3357.
14. **Moriya Y, Itoh M, Okuda S, Yoshizawa AC, Kanehisa M**. KAAS: an automatic genome annotation and pathway reconstruction server. *Nucleic Acids Res* 2007;35(suppl 2):W182-W185.
15. **Takami H, Taniguchi T, Arai W, Takemoto K, Moriya Y et al.** An automated system for evaluation of the potential functionome: MAPLE version 2.1. 0. *DNA Res* 2016;23(5):467-475.
16. **Li PE, Lo CC, Anderson JJ, Davenport KW, Bishop-Lilly KA et al.** Enabling the democratization of the genomics revolution with a fully integrated web-based bioinformatics platform. *Nucleic Acids Res* 2017;45(1):67-80.
17. **Botelho J, Grosso F, Quinteira S, Mabrouk A, Peixe L**. The complete nucleotide sequence of an IncP-2 megaplasmid unveils a mosaic architecture comprising a putative novel blaVIM-2-harbouring transposon in *Pseudomonas aeruginosa*. *J Antimicrob Chemother* 2017;72(8):2225-2229.
18. **Xiong J, Alexander DC, Ma JH, Deraspe M, Low DE et al.** Complete sequence of pOZ176, a 500-kilobase IncP-2 plasmid encoding IMP-9-mediated carbapenem resistance, from outbreak isolate *Pseudomonas aeruginosa* 96. *Antimicrob Agents Chemother* 2013;57(8):3775-3782.
19. **Naughton S, Parker D, Seemann T, Thomas T, Turnbull L et al.** *Pseudomonas aeruginosa* AES-1 exhibits increased virulence gene expression during chronic infection of cystic fibrosis lung. *PLoS One* 2011;6(9):e24526.
20. **Rau MH, Marvig RL, Ehrlich GD, Molin S, Jelsbak L**. Deletion and acquisition of genomic content during early stage adaptation of *Pseudomonas aeruginosa* to a human host environment. *Environ Microbiol* 2012;14(8):2200-2211.
21. **Winstanley C, Langille MG, Fothergill JL, Kukavica-Ibrulj I, Paradis-Bleau C et al.** Newly introduced genomic prophage islands are critical determinants of in vivo competitiveness in the Liverpool Epidemic Strain of *Pseudomonas aeruginosa*. *Genome Res* 2009;19(1):12-23.
22. **Stover CK, Pham XQ, Erwin AL, Mizoguchi SD, Warrener P et al.** Complete genome sequence of *Pseudomonas aeruginosa* PAO1, an opportunistic pathogen. *Nature* 2000;406:959-964.
23. **Lee DG, Urbach JM, Wu G, Liberati NT, Feinbaum RL et al.** Genomic analysis reveals that *Pseudomonas aeruginosa* virulence is combinatorial. *Genome biology* 2006;7(10):R90.

24. **Mathee K, Narasimhan G, Valdes C, Qiu X, Matewish JM et al.** Dynamics of *Pseudomonas aeruginosa* genome evolution. *Proceedings of the National Academy of Sciences* 2008;105(8):3100-3105.

25. **O'Carroll MR, Syrmis MW, Wainwright CE, Greer RM, Mitchell P et al.** Clonal strains of *Pseudomonas aeruginosa* in paediatric and adult cystic fibrosis units. *Eur Respir J* 2004;24(1):101-106.

26. **Bradbury RS, Roddam LF, Merritt A, Reid DW, Champion AC**. Virulence gene distribution in clinical, nosocomial and environmental isolates of *Pseudomonas aeruginosa*. *J Med Microbiol* 2010;59(8):881-890.

27. **Totten PA, Lory S**. Characterization of the type a flagellin gene from Pseudomonas aeruginosa PAK. *J Bacteriol* 1990;172(12):7188-7199.

28. **Toussaint B, Delic-Attree I, Vignais PM**. *Pseudomonas aeruginosa* contains an IHF-like protein that binds to the algD promoter. *Biochem Biophys Res Commun* 1993;196(1):416-421.

29. **Cramer N., Wiehlmann L., Ciofu O., Tamm S., Høiby N. et al.** Molecular epidemiology of chronic *Pseudomonas aeruginosa* airway infections in cystic fibrosis. *PLoS ONE* 2012;7(11):e50731. doi:50710.51371/journal.pone.0050731

30. **Stewart RMK, Wiehlmann L, Ashelford KE, Preston SJ, Frimmersdorf E et al.** Genetic Characterization Indicates that a Specific Subpopulation of *Pseudomonas aeruginosa* Is Associated with Keratitis Infections. *J Clin Microbiol* 2011;49(3):993-1003.

31. **Pirnay J, Bilocq F, Pot B, Cornelis P, Zizi M et al.** Pseudomonas aeruginosa population structure revisited. *PLoS ONE* 2009;4(11):e7740.

32. **Rakhimova E, Wiehlmann L, Brauer AL, Sethi S, Murphy TF et al.** *Pseudomonas aeruginosa* Population Biology in Chronic Obstructive Pulmonary Disease. *J Infect Dis* 2009;200(12):1928-1935.

33. **Karna SR, Chen T, Chen P, Peacock TJ, Abercrombie JJ et al.** Genome Sequence of a Virulent *Pseudomonas aeruginosa* Strain, 12-4-4 (59), Isolated from the Blood Culture of a Burn Patient. *Genome announcements* 2016;4(2):e00079-00016.

34. **Boyle B, Fernandez L, Laroche J, Kukavica-Ibrulj I, Mendes CM et al.** Complete genome sequences of three *Pseudomonas aeruginosa* isolates with phenotypes of polymyxin B adaptation and inducible resistance. *J Bacteriol* 2012;194(2):529-530.

35. **Ichise Y-k, Kosuge T, Uwate M, Nakae T, Maseda H**. Complete genome sequence of *Pseudomonas aeruginosa* strain 8380, isolated from the human gut. *Genome announcements* 2015;3(3):e00520-00515.

36. **Feng Y, Jonker MJ, Moustakas I, Brul S, ter Kuile BH**. Dynamics of mutations during development of resistance by *Pseudomonas aeruginosa* against five antibiotics. *Antimicrob Agents Chemother* 2016;60(7):4229-4236.

37. **Valot B, Rohmer L, Jacobs MA, Miller SI, Bertrand X et al.** Comparative genomic analysis of two multidrug-resistant clinical isolates of ST395 epidemic strain of *Pseudomonas aeruginosa* obtained 12 years apart. *Genome announcements* 2014;2(3):e00515-00514.

38. **Nakano K, Terabayashi Y, Shiroma A, Shimoji M, Tamotsu H et al.** First complete genome sequence of *Pseudomonas aeruginosa* (Schroeter 1872) Migula 1900 (DSM 50071T), determined using PacBio single-molecule real-time technology. *Genome announcements* 2015;3(4):e00932-00915.

39. **Fothergill JL, Walshaw MJ, Winstanley C**. Transmissible strains of *Pseudomonas aeruginosa* in cystic fibrosis lung infections. *Eur Respir J* 2012;40(1):227-238.

40. **Wu D-Q, Ye J, Ou H-Y, Wei X, Huang X et al.** Genomic analysis and temperature-dependent transcriptome profiles of the rhizosphere originating strain *Pseudomonas aeruginosa* M18. *BMC Genomics* 2011;12(1):438.

41. **Ohtsubo Y, Sato T, Kishida K, Tabata M, Ogura Y et al.** Complete genome sequence of *Pseudomonas aeruginosa* MTB-1, isolated from a microbial community enriched by the technical formulation of hexachlorocyclohexane. *Genome announcements* 2014;2(1):e01130-01113.

42. **Miyoshi-Akiyama T, Kuwahara T, Tada T, Kitao T, Kirikae T**. Complete genome sequence of highly multidrug-resistant *Pseudomonas aeruginosa* NCGM2. S1, a representative strain of a cluster endemic to Japan. *J Bacteriol* 2011;193(24):7010-7010.

43. **Tada T, Miyoshi-Akiyama T, Shimada K, Shiroma A, Nakano K et al.** A Carbapenem-Resistant *Pseudomonas aeruginosa* Isolate Harboring Two Copies of blaIMP-34 Encoding a Metallo-β-Lactamase. *PloS one* 2016;11(4):e0149385.

44. **Lu S, Le S, Li G, Shen M, Tan Y et al.** Complete genome sequence of *Pseudomonas aeruginosa* PA1, isolated from a patient with a respiratory tract infection. *Genome announcements* 2015;3(6):e01453-01415.

45. **Li G, Lu S, Shen M, Le S, Tan Y et al.** Complete genome sequence of *Pseudomonas aeruginosa* phage-resistant variant PA1RG. *Genome announcements* 2016;4(1):e01761-01715.
46. **Roy PH, Tetu SG, Larouche A, Elbourne L, Tremblay S et al.** Complete genome sequence of the multiresistant taxonomic outlier *Pseudomonas aeruginosa* PA7. *PloS one* 2010;5(1):e8842.
47. **Jeukens J, Boyle B, Bianconi I, Kukavica-Ibrulj I, Tümmler B et al.** Complete genome sequence of persistent cystic fibrosis isolate *Pseudomonas aeruginosa* strain RP73. *Genome announcements* 2013;1(4):e00568-00513.
48. **Miller CL, Chen T, Chen P, Leung KP**. Genome sequence of highly virulent *Pseudomonas aeruginosa* strain VA-134, isolated from a burn patient. *Genome announcements* 2016;4(1):e01662-01615.
49. **Murugan N, Malathi J, Umashankar V, Madhavan H**. Unraveling genomic and phenotypic nature of multidrug-resistant (MDR) *Pseudomonas aeruginosa* VRFPA04 isolated from keratitis patient. *Microbiol Res* 2016;193:140-149.
50. **Chan K-G, Yin W-F, Lim YL**. Complete genome sequence of *Pseudomonas aeruginosa* strain YL84, a quorum-sensing strain isolated from compost. *Genome announcements* 2014;2(2).
51. **Déraspe M, Alexander DC, Xiong J, Ma JH, Low DE et al.** Genomic analysis of *Pseudomonas aeruginosa* PA96, the host of carbapenem resistance plasmid pOZ176. *FEMS Microbiol Lett* 2014;356(2):212-216.
52. **Roy AS, Baruah R, Gogoi D, Borah M, Singh AK et al.** Draft genome sequence of *Pseudomonas aeruginosa* strain N002, isolated from crude oil-contaminated soil from Geleky, Assam, India. *Genome announcements* 2013;1(1):e00104-00112.
53. **Liu H, Liang R, Tao F, Ma C, Liu Y et al.** Genome sequence of *Pseudomonas aeruginosa* strain SJTD-1, a bacterium capable of degrading long-chain alkanes and crude oil. *J Bacteriol* 2012;194(17):4783-4784.
54. **Chugani S, Kim BS, Phattarasukol S, Brittnacher MJ, Choi SH et al.** Strain-dependent diversity in the *Pseudomonas aeruginosa* quorum-sensing regulon. *Proceedings of the National Academy of Sciences* 2012;109(41):E2823-E2831.
55. **Stewart L, Ford A, Sangal V, Jeukens J, Boyle B et al.** Draft genomes of 12 host-adapted and environmental isolates of *Pseudomonas aeruginosa* and their positions in the core genome phylogeny. *Pathogens and disease* 2014;71(1):20-25.
56. **Gao C, Hu C, Ma C, Su F, Yu H et al.** Genome sequence of the lactate-utilizing *Pseudomonas aeruginosa* strain XMG. *J Bacteriol* 2012;194(17):4751-4752.

Table S1: Collection of industrial and other *P. aeruginosa* strains assembled and analysed in this study

Strain Name	Type of strain	Isolation source and comments	Isolation date	Isolation region (country)	Isolation region (continent)	Reference	GenBank accession number	Analysis performed						
								Genome comparison	RAPD	AT	MLST, rMLST, wgMLST	Phenotypic testing	IPARP	SPAdes genome assembly
AES-1R-2482	Clinical	Australian epidemic strain isolated from sputum of a 14 month old infant with cystic fibrosis		Australia		[19]	CP013680	■				■	■	
DK2	Clinical	CF sputum		Denmark	Europe	[20]	CP003149	■			■	■	■	
LESB58	Clinical	CF sputum, Liverpool epidemic strain	1988	UK	Europe	[21]	FM209186	■			■	■	■	
PAO1	Clinical	Wound				[22]	AE004091	■			■	■	■	
UCBPP-PA14	Clinical	Burn patient				[23]	CP000438	■		■	■	■	■	
C3719	Clinical	CF; Manchester epidemic strain		UK	Europe	[24]	AAKV00000000				■	■	■	
AUS23	Clinical	CF; Austrialian epidemic strain 2 (AES-2)		Australia		[25]	Not available					■	■	
AUS52	Clinical	CF; Austrialian epidemic strain 3 (AES-3)		Australia		[26]	Not available					■	■	
PAK	Clinical	Widely studied; expresses pili, flagella and glycosylation islands				[27]	GCA_000568855.1					■	■	
CHA	Clinical	Detailed phenotypic characterisation available				[28]	Not available					■	■	
NN2	Clinical	Detailed phenotypic characterisation available; Clone C		Germany	Europe	[29]	LT883143					■	■	
39016	Clinical	Keratitis eye isolate		UK	Europe	[30]	NZ_CM001020					■	■	
1709-12	Clinical	Multidrug resistance; Serotype O12	2004	Belgium	Europe	[31]	NZ_LZQH00000000					■	■	
Mi 162	Clinical	Multidrug resistance; Serotype O11	1997	Michigan, USA	North America	[31]	Not available					■	■	
Jpn 1563	Environmental	Lake water	2003	Japan	Asia	[31]	Not available					■	■	
LMG 14084	Environmental	Water	1960-1964	Romania	Europe	[31]	Not available					■	■	
Pr335	Clinical/ Environmental	Nosocomial environment	1997	Czech Republic	Europe	[31]	Not available					■	■	
CPHL 9433	Environmental	Tobacco plant		Phillipines	Asia	[31]	Not available					■	■	
RP1	Clinical	CF		Germany	Europe	[29]	LNBU00000000					■	■	
57P31PA	Clinical	Chronic obstructive pulmonary disease		USA	North America	[32]	Not available					■	■	
39177	Clinical	Keratitis		UK	Europe	[30]	Not available					■	■	
12-4-4.59	Clinical	Blood culture of a burn patient				[33]	CP013696	■						

19BR	Clinical	Collected as part of a Brazilian surveillance study between 2002 and 2004	2002-2004	Brazil	South America	[34]	AFXJ00000000							
213BR	Clinical	Collected as part of a Brazilian surveillance study between 2002 and 2004	2002-2004	Brazil	South America	[34]	AFXK00000000							
8380-3922	Clinical	Human gut				[35]	AP014839							
ATCC-15692	Clinical	Infected wound				Not published	CP017149							
ATCC-27853	Clinical	Unknown				[36]	CP015117							
B136-33	Clinical	Infant with community acquired diarrhoea				Not Published	CP004061							
BAMC-07-48	Clinical	Combat injury wound				(Sanjar et al. 2016)	CP015377							
Carb01-63	Clinical	Unknown				Not published	CP011317							
DHS01	Clinical	Nose of a patient				[37]	CP013993							
DN1	Environmental	Soil, China		China		Not published	CP017099							
DSM-50071	Clinical	Hospital, Japan		Japan	Asia	[38]	CP012001							
F9676	Environmental	Diseased rice, China		China	Asia	Not published	CP012066							
F22031	Clinical	Pubic bone				Not published	CP007399							
FA-HZ1	Environmental	Wastewater, dibenzufuran_degrading bacterium				Not published	CP017353							
FRD1	Clinical	CF sputum				Not published	CP010555							
IOMTU-133	Clinical	Female participant in the dbGaP microbiome study				Not published	AP017302							
F9670	Clinical	Unknown				Not published	CP008873							
F23197	Clinical	Unknown				Not published	CP008856							
F30658	Clinical	Unknown				Not published	CP008857							
F63912	Clinical	Unknown				Not published	CP008858							
H5708	Clinical	Unknown				Not published	CP008859							
H27930	Clinical	Unknown				Not published	CP008860							
H47921	Clinical	Unknown				Not published	CP008861							
M1608	Clinical	Unknown				Not published	CP008862							
M37351	Clinical	Unknown				Not published	CP008863							
S86968	Clinical	Unknown				Not published	CP008865							
T38079	Clinical	Unknown				Not published	CP008866							
T52373	Clinical	Unknown				Not published	CP008867							

T63266	Clinical	Unknown				Not published	CP008868							
W16407	Clinical	Unknown				Not published	CP008869							
W36662	Clinical	Unknown				Not published	CP008870							
W45909	Clinical	Unknown				Not published	CP008871							
W60856	Clinical	Unknown				Not published	CP008864							
X78812	Clinical	Unknown				Not published	CP008872							
LES431	Clinical	CF sputum, Liverpool epidemic strain		UK	Europe	[39]	CP006937							
M18	Environmental	Plant isolate, China		China	Asia	[40]	CP002496							
MTB-1	Environmental	Hexachlorocyclohexane contaminated soil				[41]	CP006853							
N17-1	Environmental	Soil				Not published	CP014948							
NCGM2.S1	Clinical	Isolated from a hospital in Japan		Japan	Asia	[42]	AP012280							
NCGM257	Clinical	Urine, Japan				Not published	AP014651							
NCGM1900	Clinical	Urinary catheter				Not published	AP014622							
NCGM1984	Clinical	Urinary catheter				[43]	AP014646							
NCTC10332	Clinical	Unknown				Not published	LN831024							
D1	Clinical	Clinical isolate from ventilator associated pneumonia patient				Not published	CP012585							
D2	Clinical	Clinical isolate from ventilator associated pneumonia patient				Not published	CP012578							
D5	Clinical	Clinical isolate from ventilator associated pneumonia patient				Not published	CP012579							
D9	Clinical	Clinical isolate from ventilator associated pneumonia patient				Not published	CP012580							
D16	Clinical	Clinical isolate from ventilator associated pneumonia patient				Not published	CP012581							
D21	Clinical	Clinical isolate from ventilator associated pneumonia patient				Not published	CP012582							
D22	Clinical	Clinical isolate from ventilator associated pneumonia patient				Not published	CP012583							
D25	Clinical	Clinical isolate from ventilator associated pneumonia patient				Not published	CP012584							
PA1	Clinical	Respiratory tract infection				[44]	CP004054							
PA1R	Clinical	Respiratory tract infection				[44]	CP004055							
PA1RG	Clinical/environmental	Hospital sewage				[45]	CP012679							

PA7	Clinical	Non_respiratory clinical isolate				[46]	CP000744							
PA121617	Clinical	CF Sputum				Not published	CP016214							
PACS2	Clinical	CF Sputum				Not published	AAQW01000001							
RP73	Clinical	CF Sputum				[47]	CP006245							
SCV20265	Clinical	CF Lung				(Eckweiler et al. 2014)	CP006931							
USDA-ARS-USMARC-41639	Environmental	Nasopharynx of a cow in Kansas, USA		Kansas, USA	North America	Not published	CP013989							
VA-134	Clinical	Skin wound of burn human patient				[48]	CP013245							
VRFPA04	Clinical	Corneal button from patient with corneal keratitis				[49]	CP008739							
YL84	Environmental	Compost				[50]	CP007147							
PA96	Clinical	Clinical isolate from Guangzhou, China		China	Asia	[51]	CP007224							
S04-90	Environmental	Microbial mat material				Not published	NZ_CP011369							
N002	Environmental	Crude oil contaminated soil				[52]	ALBV00000000							
SJTD-1	Environmental	Soil				[53]	CP015877							
ATCC-700888	Environmental	Industrial water system				[54]	AKZF00000000							
E2oS	Environmental	Soil				[55]	ASQV00000000							
MSH-3	Environmental	Environmental, Mount St. Helens		Washington, USA	North America	[55]	ASQU00000000							
ATCC-14886	Environmental	Soil				[54]	AKZD00000000							
MSH-10	Environmental	Environmental				[55]	ASWW00000000							
XMG	Environmental	Soil, China		China	Asia	[56]	AJXX00000000							
ATCC-12903 (NCTC 12903)	Clinical	Antibiotic efficacy testing reference strain; originally isolated from blood				This study	GCA_001374435.1							
RW18	Clinical	Chronic prostatitis isolate		UK	Europe	This study	GCA_001374635.1							
RW27	Clinical	CF sputum		BC, Canada	North America	This study	GCA_001373635.1							
RW30	Clinical	CF sputum		BC, Canada	North America	This study	GCA_001373875.1							
RW99	Environmental	Domestic isolate, washing machine drawer biofilm		UK	Europe	This study	GCA_001374955.1							
RW109	Industrial	Personal care product; preservative efficacy testing strain	2003		Europe	This study	GCA_900243355.1							
RW110	Industrial	Household cleaner; preservative efficacy testing strain				This study	GCA_001374115.1							
RW 113	Industrial	Household cleaner	2011	Unknown	Unknown	This study	Not available							

RW 114	Industrial	Household cleaner	2011	Unknown	Unknown	This study	Not available							
RW 115	Industrial	Household cleaner	2011	Unknown	Unknown	This study	Not available							
RW 116	Industrial	Household cleaner	2011	Unknown	Unknown	This study	Not available							
RW 117	Industrial	Household cleaner	2010		Asia	This study	Not available							
RW 118	Industrial	Household cleaner	2010		Asia	This study	Not available							
RW 119	Industrial	Household cleaner	2010		Europe	This study	Not available							
RW 120	Industrial	Household cleaner	2010		Europe	This study	Not available							
RW 121	Industrial	Household cleaner	2010		Europe	This study	Not available							
RW 122	Industrial	Household cleaner	2009		Europe	This study	Not available							
RW 123	Industrial	Household cleaner	2011		Asia	This study	Not available							
RW 124	Industrial	Household cleaner	2009		Europe	This study	Not available							
RW 125	Industrial	Household cleaner	2009		Europe	This study	Not available							
RW 126	Industrial	Household cleaner	2009		Europe	Not published	Not available							
RW 127	Industrial	Household cleaner	2010		Asia	This study	Not available							
RW 128	Industrial	Household cleaner	2010		Europe	This study	Not available							
RW 129	Industrial	Household cleaner	2010		Europe	This study	Not available							
RW130	Industrial	Household cleaner	2010		Europe	This study	GCA_001374355.1							
RW131	Industrial	Household cleaner	2010		Europe	This study	GCA_001374455.1							
RW 132	Industrial	Household cleaner	2010		Europe	This study	Not available							
RW 133	Industrial	Household cleaner	2010		Asia	This study	Not available							
RW 134	Industrial	Household cleaner	2010		Asia	This study	Not available							
RW 135	Industrial	Household cleaner	2010		Asia	This study	Not available							
RW 136	Industrial	Household cleaner	2010		Asia	This study	Not available							
RW 137	Industrial	Household cleaner	2010		Europe	This study	Not available							
RW138	Industrial	Household cleaner	2001	Unknown	Unknown	This study	GCA_001374655.1							
RW 139	Industrial	Household cleaner	2001	Unknown	Unknown	This study	Not available							
RW 140	Industrial	Household cleaner	Unknown	Unknown	Unknown	This study	Not available							
RW 143	Industrial	Household cleaner	Unknown	Unknown	Unknown	This study	Not available							
RW 144	Industrial	Household cleaner	Unknown	Unknown	Unknown	This study	Not available							

RW 145	Industrial	Household cleaner	2010	Unknown	Unknown	This study	Not available							
RW146	Industrial	Household cleaner	2004		Europe	This study	GCA_001373655.1							
RW 147	Industrial	Personal care product	Unknown	Unknown	Unknown	This study	Not available							
RW 148	Industrial	Personal care product	Unknown	Unknown	Unknown	This study	Not available							
RW149	Industrial	Personal care product	Unknown	Unknown	Unknown	This study	GCA_001373895.1							
RW 150	Industrial	Personal care product	Unknown	Unknown	Unknown	This study	Not available							
RW168.2	Industrial	Laundry liquid	Unknown	Unknown	Unknown	This study	GCA_001375215.1							
RW172	Industrial	Household cleaner	2009	Unknown	Unknown	This study	GCA_001374135.1							
RW 173	Industrial	Household cleaner	2011		Asia	This study	Not available							
RW 174	Industrial	Household cleaner	2010		Europe	This study	Not available							
RW 175	Industrial	Household cleaner	2010		Asia	This study	Not available							
RW176	Industrial	Household cleaner	2010		Asia	This study	GCA_001374375.1							
RW 177	Industrial	Household cleaner	2010		Asia	This study	Not available							
RW 178	Industrial	Household cleaner	2010		Asia	This study	Not available							
RW 179	Industrial	Household cleaner	2010		Asia	This study	Not available							
RW 180	Industrial	Household cleaner	Unknown	Unknown	Unknown	This study	Not available							
RW 181	Industrial	Household cleaner	Unknown	Unknown	Unknown	This study	Not available							
RW 182	Industrial	Household cleaner	Unknown	Unknown	Unknown	This study	Not available							
RW184	Industrial	Household cleaner	Unknown	Unknown	Unknown	This study	GCA_001373595.1							
RW 185	Industrial	Household cleaner	Unknown	Unknown	Unknown	This study	Not available							
RW 187	Industrial	Personal care product	Unknown	Unknown	Unknown	This study	Not available							
RW 188	Industrial	Personal care product	Unknown	Unknown	Unknown	This study	Not available							
RW 189	Industrial	Personal care product	Unknown	Unknown	Unknown	This study	Not available							
RW 190	Industrial	Household cleaner	Unknown	Unknown	Unknown	This study	Not available							
RW 191	Industrial	Household cleaner	Unknown	Unknown	Unknown	This study	Not available							
RW192	Industrial	Household cleaner	Unknown	Unknown	Unknown	This study	GCA_001374675.1							
RW 193	Industrial	Personal care product	2003	Unknown	Unknown	This study	Not available							
RW 194	Industrial	Household cleaner	2011		South America	This study	Not available							
RW 195	Industrial	Household cleaner	2006	Unknown	Unknown	This study	Not available							

RW199	Industrial	Metal working fluid product	Unknown	Unknown	Unknown	This study	GCA_001374995.1							
RW200	Industrial	Timber care product	Unknown	Unknown	Unknown	This study	GCA_001375235.1							
RW202	Industrial	Household cleaner	2012	Europe	Europe	This study	GCA_001374155.1							
RW204	Industrial	Household cleaner	2012	Unknown	Unknown	This study	GCA_001374395.1							
ATCC-9027 (RW 151)	Reference strain (clinical)	Reference strain used in industrial testing; isolated from an outer ear infection	Unknown	Unknown	Unknown	This study	GCA_001374975.1							
ATCC-13388 (RW196)	Reference strain (unknown)	Reference strain used in industrial testing (ISO 846C)	Unknown	Unknown	Unknown	This study	GCA_001373675.1							
ATCC 10145 (RW197)	Reference strain (unknown)	Reference strain used in industrial testing	Unknown	Unknown	Unknown	This study	Not available							
ATCC-15442 (RW 198)	Reference strain (environmental)	Reference strain used in industrial testing; isolated from an animal room water bottle	Unknown	Unknown	Unknown	This study	GCA_001373915.1							

Table S2. ArrayTube (AT) genotypes of industrial and reference testing *P. aeruginosa* strains

Strain (RW#)	Other names	Isolation source/comment	16 digit code	Hexadecimal code (AT-genotype)	Database match and comments
**109***	-	IND; PC; North America; isolated 2003; Strain used in preservative efficacy testing	0110-1100-0010-0010	6C22	Clone Y, previously associated with CF, CLIN, ENV
**110***	-	IND; HC; Europe; isolated 2003; Strain used in preservative efficacy testing	1111-0100-0110-1001	F469	Clone D, previously associated with CF, CLIN, COPD, KER, ENV
**130***	-	IND; HC; Europe; isolated 2010	1010 -1111-1010-1010	AFAA	Genotype previously associated with CF
**131***	-	IND; HC; Europe; isolated 2010	1010 -1111-1010-1010	AFAA	Genotype previously associated with CF
**138***	-	IND; HC; Europe; isolated 2001	0110-1101-1001-0010	6D92	Clone H, previously associated with CF, CLIN, COPD, ENV
**146***	-	IND; HC; Europe; isolated 2004	1010 -1111-1010-1010	AFAA	Genotype previously associated with CF
**149***	-	IND; PC; origin unknown; isolated 2003	0010-1100-0101-0010	2C52	Novel type
**168.2**	-	IND; LL; origin and isolation date unknown; typing unsuccessful	-	-	-
**172***	-	IND; HC; Asia; isolated 2009	1110-0100-0010-1001	E429	Clone B, previously associated with CF, CLIN, COPD, KER, ENV
**176***	-	IND; HC; Asia; isolated 2010	0010-1100-0101-0010	2C52	Novel type
**184***	-	IND; HC; Europe; isolated 2006	0001-1011-1010-1010	1BAA	Novel type
**192***	-	IND; SC; origin unknown; isolated 2012	0110-1100-0010-0010	6C22	Clone Y, previously associated with CF, CLIN, ENV
**199***	-	IND; MWF; origin and isolation date unknown	0010-1111-1010-1010	2FAA	Genotype associated with CF and ENV
**200***	-	IND; TC; origin and isolation date unknown	0010-1011-1001-0010	2B92	Novel type
**202***	-	IND; HC; Europe isolated 2012	1011-0100-0110-1001	B469	Genotype associated with CLIN, KER
**204***	-	IND; HC; isolated 2012	0010-1100-1001-1010	2C9A	Genotype associated with CF, ENV
**151***	ATCC 9027	CLIN; reference strain used in industrial testing; originally isolated from an outer ear infection	0101-1100-0001-1010	5C1A	Novel type
**196***	ATCC 13388; NCTC 8060	Origin unknown; reference strain used in industrial testing (ISO 846C)	0000-1011-1001-0010	0B92	Clone X, previously associated with ENV
**198***	ATCC 15442	ENV; reference strain used in industrial testing, originally isolated from an animal room water bottle	0000-0101-1001-1010	059A	Genotype previously associated with CF, CLIN

Abbreviations: CLIN, clinical; CF, cystic fibrosis; COPD, chronic obstructive pulmonary disease; KER, keratitis; ENV, environmental; IND, industrial; HC, household cleaner; PC, personal care product; LL, laundry liquid; MWF, metal working fluid; TC, timber care. *AT-genotypes for these strains (as strains associated with industry) are highlighted in Figure 2

Table S3. MLST allele and Sequence Type (ST) designations for industrial and reference testing *P. aeruginosa* strains

Strain	Isolation source	MLST loci							ST	ST isolation sources[a]
		acs	*aro*	*gua*	*mut*	*nuo*	*pps*	*trp*		
**RW109**	IND; PC; North America; isolated 2003; Strain used in preservative efficacy testing	17	5	5	4	4	4	3	111	CF, CLIN, ENV, OTHER
**RW110**	IND; HC; Europe; isolated 2003; Strain used in preservative efficacy testing	5	141	65	151	1	33	50	2729	Novel
**RW130**	IND; HC; Europe; isolated 2010	15	48	20	142	4	7	7	2730	Novel
**RW131**	IND; HC; Europe; isolated 2010	15	48	20	142	4	7	7	2730	Novel
**RW138**	IND; HC; Europe; isolated 2001	17	5	5	4	4	4	3	111	CF, CLIN, ENV, OTHER
**RW146**	IND; HC; Europe; isolated 2004	15	48	20	142	4	7	7	2730	Novel
**RW149**	IND; PC; origin unknown; isolated 2003	1	5	26	3	1	10	3	1342	OTHER
**RW168.2**	IND; LL; origin and isolation date unknown	40	5	3	162	73	75	2	2733	Novel
**RW172**	IND; HC; Asia; isolated 2009	13	8	9	3	1	6	9	316	CF, CLIN, ENV, OTHER
**RW176**	IND; HC; Asia; isolated 2010	1	5	26	3	1	10	3	1342	OTHER
**RW184**	IND; HC; Europe; isolated 2006	28	5	36	3	3	13	7	155	CF, ENV, OTHER
**RW192**	IND; HC; origin unknown; isolated 2012	17	5	5	4	4	4	3	111	CF, CLIN, ENV, OTHER
**RW199**	IND; MWF; origin and isolation date unknown	17	22	11	3	3	15	3	800	CF
**RW200**	IND; TC; origin and isolation date unknown	6	5	6	5	4	4	7	641	CLIN
**RW202**	IND; HC; Europe isolated 2012	5	141	65	151	1	33	50	2729	Novel
**RW204**	IND; HC; isolated 2012	6	5	5	3	3	13	1	645	CF, CLIN,ENV
**RW151; ATCC 9027**	CLIN; reference strain used in industrial testing; originally isolated from an outer ear infection	23	5	12	30	1	4	7	1105	CF
**RW196; ATCC 13388**	Origin unknown; reference strain used in industrial testing (ISO 846C)	17	5	12	3	14	4	7	244	CF, CLIN, ENV, OTHER
**RW198; ATCC 15442**	ENV; reference strain used in industrial testing, originally isolated from an animal room water bottle	6	28	4	3	3	4	7	252	CF, CLIN, ENV, OTHER

Abbreviations: CLIN, clinical; ENV, environmental; IND, industrial; HC, household cleaner; PC, personal care product; LL, laundry liquid; MWF, metal working fluid; TC, timber care;

[a]Isolation source from PubMLST database; coloured cells are STs found more than once

Table S4. *P. aeruginosa* tolerance of the isothiazolinone preservatives MIT, CITMIT and BIT

Strain	Other names	Isolation source	MIC values (%)					
			MIT		CITMIT		BIT	
			Median	Range[a]	Median	Range	Median	Range
LESB58	-	CF	0.0000781	-	0.0002345	0.000156-0.000313	0.000313	-
C3719	-	CF	0.000313	-	0.0007815	0.00313-0,00125	0.000625	-
DK2	-	CF	0.001875	0.00125-0.0025	0.0009375	0.000625-0.000125	0.005	-
AES-1R	-	CF	0.0025	-	0.0007815	0.00313-0.00125	0.005	-
AUS23	-	CF	0.000313	-	0.0007815	0.00313-0.00125	0.00375	0.0025-0.005
AUS52	-	CF	0.000625	-	0.000313	-	0.0015625	0.000625-0.0025
PAO1	-	CLIN	0.001875	0.00125-0.005	0.0009375	0.000625-0.000125	0.0075	0.005-0.01
UCBPP-PA14	-	CLIN	0.00125	-	0.0009375	0.000625-0.000125	0.005	-
PAK	-	CLIN	0.00125	0.00125-0.0025	0.0007815	0.000625-0.000125	0.00375	0.0025-0.005
CHA	-	CF	0.0025	-	0.0009375	0.000625-0.000125	0.005	-
NN2	-	CF	0.001875	0.00125-0.005	0.0007815	0.000625-0.00025	0.005	-
39016	-	CLIN	0.0025	-	0.0009375	0.00313-0.00125	0.01	-
1709-12	-	CF	0.0025	0.0025-0.005	0.0007815	0.00313-0.00125	0.01	-
Mi 162	-	CLIN	0.00125	-	0.0007815	0.00313-0.00125	0.005	0.005-0.01
Jpn 1563	-	ENV	0.00375	0.0025-0.005	0.0015625	0.000625-0.00025	0.01	-
LMG 14084	-	ENV	0.0025	-	0.0009375	0.000625-0.00025	0.005	-
Pr335	-	ENV	0.001875	0.00125-0.0025	0.0009375	0.000625-0.00025	0.00375	0.0025-0.005
CPHL 9433	-	ENV	0.00375	0.0025-0.005	0.0009375	0.000625-0.00025	0.01	0.005-0.01
RP1	-	CF	0.00375	0.0025-0.005	0.0015625	0.000625-0.00025	0.005	-
57P31PA	-	CLIN	0.0025	-	0.0015625	0.000625-0.000125	0.01	-
39177	-	CLIN	0.001875	0.00125-0.0025	0.0009375	0.000625-0.000125	0.005	-
NCTC 12903; ATCC 27853	RW11	CLIN	0.00375	0.0025-0.005	0.0015625	0.000625-0.00025	0.01	0.005-0.01
RW109	-	IND; PC	0.0025	0.0025-0.005	0.001875	0.00125-0.0025	0.01	-

RW110	-	IND; HC	0.0025	0.0025-0.005	0.0015625	0.000625-0.00025	0.005	0.005-0.01
RW130	-	IND; HC	0.00375	0.0025-0.005	0.0015625	0.000625-0.00025	0.02	-
RW131	-	IND; HC	0.00375	0.0025-0.005	0.001875	0.00125-0.0025	0.02	-
RW138	-	IND; HC	0.005	-	0.0009375	0.000625-0.000125	0.04	-
RW146	-	IND; HC	0.005	-	0.0015625	0.000625-0.00025	0.02	-
RW149	-	IND; PC	0.00375	0.0025-0.005	0.0009375	0.000625-0.000125	0.01	-
ATCC 9027	RW151	CLIN	0.0025	-	0.0015625	0.000625-0.00025	0.01	-
RW172	-	IND; HC	0.0025	-	0.001875	0.00125-0.0025	0.005	-
RW176	-	IND; HC	0.005	-	0.005	-	0.02	-
RW184	-	IND; HC	0.00125	0.00125-0.0025	0.0009375	0.000625-0.00025	0.005	0.0025-0.005
RW192	-	IND; HC	0.00375	0.0025-0.005	0.0015625	0.000625-0.00025	0.04	-
ATCC 13388; NCTC 8060	RW196	UNKNOWN	0.001875	0.00125-0.0025	0.00125	0.000625-0.000125	0.00375	0.0025-0.005
ATCC 15442	RW198	ENV	0.0025	0.0025-0.005	0.0015625	0.000625-0.00025	0.005	-
RW199	-	IND; MWF	0.00125	-	0.0009375	0.000625-0.000125	0.005	-
RW200	-	IND; TC	0.0025	0.00125-0.0025	0.0015625	0.000625-0.00025	0.005	-
RW202	-	IND; HC	0.0025	-	0.0015625	0.000625-0.00025	0.01	-
RW204	-	IND; HC	0.001875	0.00125-0.0025	0.0015625	0.000625-0.0025	0.01	0.005-0.01
Median MIC (%)			0.0025		0.0009375		0.005	
MIC range (%)			0.0000781-0.005		0.0002345-0.005		0.000313-0.04	
Maximum EU regulated levels (%)[b]			0.01		0.0015		0.2[c]	

Footnotes: MIC, minimum inhibitory concentration; MIT, methylisothiazolinone; CITMIT, chloromethylisothiazolinone and methylisothiazolinone blend in the ratio 3:1; BIT, benzisothiazolinone; CF, cystic fibrosis; CLIN, clinical; ENV, environmental; IND, industrial; HC, household cleaner; PC, personal care; MWF, metal working fluid; TC, timber care. Median values are colour coded with darker shades reflecting an increase in MIC. [a]The range is not reported where the replicate values were the same for a strain; [b] EU cosmetics directive 76/768/EEC, annex VI; [c]Not permitted in the EU, manufacturer's recommended level

Table S5. *P. aeruginosa* tolerance of the preservatives phenoxyethanol, chlorhexidine and benzoic acid

Strain	Other names	Isolation source	MIC values (%)					
			PHE		CHX		BA	
			Median	Range	Median	Range	Median	Range
LESB58	-	CF	0.1171875	0.078125-0.15625	0.0046875	0.001563-0.00625	0.0125	
C3719	-	CF	0.234375	0.15625-0.3125	0.002344	0.000781-0.003125	0.0125	
DK2	-	CF	0.46875	0.3125-0.625	0.002344	0.001563-0.003125	0.05	
AES-1R	-	CF	0.234375	0.15625-0.3125	0.002344	0.000781-0.003125	0.05	
AUS23	-	CF	0.15625	0.15625-0.3125	0.001172	0.000781-0.001563	0.025	
AUS52	-	CF	0.234375	0.15625-0.3125	0.000781		0.05	
PAO1	-	CLIN	0.46875	0.3125-0.625	0.0046875	0.003125-0.00625	0.05	
UCBPP-PA14	-	CLIN	0.390625	0.015625-0.625	0.001953	0.000781-0.00625	0.05	
PAK	-	CLIN	0.46875	0.3125-0.625	0.002344	0.001563-0.003125	0.05	
CHA	-	CF	0.46875	0.3125-0.625	0.003125	0.001563-0.003125	0.05	
NN2	-	CF	0.3125	0.15625-0.3125	0.0046875	0.003125-0.00625	0.05	
39016	-	CLIN	0.46875	0.3125-0.625	0.0046875	0.003125-0.00625	0.05	
1709-12	-	CF	0.46875	0.3125-0.625	0.0046875	0.003125-0.00625	0.05	
Mi 162	-	CLIN	0.46875	0.3125-0.625	0.002344	0.001563-0.003125	0.05	
Jpn 1563	-	ENV	0.46875	0.3125-0.625	0.0046875	0.001563-0.00625	0.075	0.1 - 0.05
LMG 14084	-	ENV	0.625	0.3125-0.625	0.0039065	0.001563-0.00625	0.05	
Pr335	-	ENV	0.625	0.3125-0.625	0.00625		0.05	
CPHL 9433	-	ENV	0.46875	0.3125-0.625	0.003125		0.05	
RP1	-	CF	0.46875	0.3125-0.625	0.0046875	0.003125-0.00625	0.05	
57P31PA	-	CLIN	0.46875	0.3125-0.625	0.0046875	0.003125-0.00625	0.05	
39177	-	CLIN	0.625	0.3125-0.625	0.0046875	0.003125-0.0125	0.05	
NCTC 12903; ATCC 27853	RW11	CLIN	0.46875	0.3125-0.625	0.002344	0.001563-0.00625	0.075	0.1-0.05

RW109	-	IND; PC	0.46875	0.3125-0.625	0.0046875	0.001563-0.00625	0.05	
RW110	-	IND; HC	0.46875	0.3125-0.625	0.00625		0.05	0.1-0.05
RW130	-	IND; HC	0.625	-	0.002344	0.001563-0.003125	0.05	0.05
RW131	-	IND; HC	0.46875	0.3125-0.625	0.002344	0.001563-0.003125	0.05	0.05
RW138	-	IND; HC	1.25	1.25-2.5	0.00058575	0.000391- 0.001563	0.05	0.05
RW146	-	IND; HC	0.46875	0.3125-0.625	0.002344	0.001563-0.003125	0.05	0.1-0.05
RW149	-	IND; PC	0.625	0.625	0.002344	0.001563-0.003125	0.05	0.05
ATCC 9027	RW151	CLIN	0.46875	0.3125-0.625	0.0046875	0.003125-0.00625	0.05	0.05
RW172	-	IND; HC	0.46875	0.3125-0.625	0.0046875	0.003125-0.00625	0.05	0.05
RW176	-	IND; HC	0.46875	0.3125-0.625	0.002344	0.001563-0.003125	0.05	0.05
RW184	-	IND; HC	0.234375	0.15625-0.625	0.000781		0.05	0.05
RW192	-	IND; HC	0.46875	0.3125-0.625	0.0046875	0.003125-0.00625	0.05	0.05
ATCC 13388; NCTC 8060	RW196	UNKNOWN	0.46875	0.3125-0.625	0.001172	0.000781-0.001563	0.05	0.05
ATCC 15442	RW198	ENV	0.46875	0.3125-0.625	0.00625	0.003125-0.0125	0.05	0.05
RW199	-	IND; MWF	0.625	0.625	0.001172	0.000781-0.003125	0.05	0.05
RW200	-	IND; TC	0.46875	0.3125-0.625	0.0046875	0.003125-0.00625	0.05	0.05
RW202	-	IND; HC	0.46875	0.3125-0.625	0.00625		0.05	0.05
RW204	-	IND; HC	0.46875	0.3125-0.625	0.0046875	0.003125-0.0125	0.05	0.05
Median MIC (%)			0.46875		0.00351575		0.05	
MIC range (%)			0.1171875-1.25		0.00058575-0.00625		0.0125-0.075	
Maximum EU regulated levels (%)[b]			1		0.3		0.5	

Footnotes: MIC, minimum inhibitory concentration; PHE, phenoxyethanol; CHX, chlorhexidine; BA, benzoic acid at pH5; CF, cystic fibrosis; CLIN, clinical; ENV, environmental; IND, industrial; HC, household cleaner; PC, personal care; MWF, metal working fluid; and TC, timber care. RW strains were from the RW collection held at Cardiff University. Median values are colour coded with darker shades reflecting an increase in MIC. [a]The range is not reported where the replicate values were the same for a strain; [b] EU cosmetics directive 76/768/EEC, annex VI

Table S6. Growth parameters of *P. aeruginosa* strains in liquid culture after 24 hours growth

Strain	Other names	Source	Lag phase (hrs)	Growth rate (hrs^{-1})	Log_{10} Max OD (420-580)
C3719	-	CF	4.45	0.02	0.19
DK2	-	CF	4.99	0.07	0.36
AES-1R	-	CF	6.65	0.02	0.30
AUS23	-	CF	3.92	0.02	0.19
AUS52	-	CF	5.51	0.01	0.16
PAO1	-	CLIN	5.14	0.07	0.37
UCBPP-PA14	-	CLIN	3.62	0.02	0.25
PAK	-	CLIN	3.69	0.03	0.35
CHA	-	CF	4.84	0.04	0.34
NN2	-	CF	5.68	0.12	0.38
39016	-	CLIN	6.79	0.06	0.37
1709-12	-	CF	5.87	0.07	0.38
Mil 162	-	CLIN	2.61	0.02	0.24
Jpn 1563	-	ENV	6.20	0.05	0.35
LMG 14084	-	ENV	5.76	0.09	0.35
Pr335	-	ENV	4.88	0.10	0.34
CPHL 9433	-	ENV	5.53	0.13	0.37
RP1	-	CF	4.67	0.08	0.37
57P31PA	-	CLIN	4.83	0.05	0.39
39177	-	CLIN	4.98	0.10	0.34
NCTC 12903; ATCC 27853	RW11	CLIN	5.18	0.11	0.32
RW109	-	IND; PC	5.24	0.06	0.38
RW110	-	IND; HC	5.21	0.08	0.36
ATCC 9027	RW151	CLIN	4.11	0.08	0.39
RW172	-	IND; HC	8.06	0.07	0.34
RW176	-	IND; HC	6.66	0.08	0.31
RW184	-	IND; HC	2.95	0.02	0.24
RW192	-	IND; HC	7.12	0.07	0.37
ATCC 15442	RW198	ENV	4.94	0.12	0.40
RW199	-	IND; MWF	6.65	0.08	0.37
RW200	-	IND; TC	5.74	0.08	0.36
RW202	-	IND; HC	5.33	0.07	0.35
RW204	-	IND; HC	6.89	0.08	0.37

Footnotes: CF, cystic fibrosis; CLIN, clinical, ENV, environmental; IND, industrial; HC, household cleaner; PC, personal care cosmetic; MWF, metal working fluid; TC, timber care. Strains LESB58, RW130, RW131, RW138, RW146, RW149 and RW196 were excluded from the analysis as they produced growth curves that could not be accurately modelled by the grofit package in R statistical software.

Table S7. Swimming, swarming and twitching motilities of *P. aeruginosa*

Strain	Other names	Source	Swimming diameter (mm)	Swarming diameter (mm)		Twitching diameter (mm)
				LB 0.5% agar	BSM-G 0.5% agar	
LESB58		CF	17.5	-	-	-
C3719		CF	-	-	-	-
DK2		CF	53.5	19	12.5	12
AES-1R		CF	51	-	-	-
AUS23		CF	52.5	-	-	9
AUS52		CF	-	-	-	-
PAO1		CLIN	60.5	28	18.5	23.5
UCBPP-PA14		CLIN	62.5	24	16.5	11
PAK		CLIN	60	12.5	10	17
CHA		CF	80	25.5	18	19.5
NN2		CF	61	12	10	20.5
39016		CLIN	75	22.5	11	30.5
1709-12		CF	29.5	7.5	-	-
Mi 162		CLIN	62.5	-	-	-
Jpn 1563		ENV	25	16.5	15	38
LMG 14084		ENV	58.5	14.5	-	-
Pr335		ENV	53.5	16	11.5	18.5
CPHL 9433		ENV	66	13.5	7.5	11.5
RP1		CF	65	26	8.5	15.5
57P31PA		CLIN	68.5	31	14	14
39177		CLIN	48.5	15.5	8	19.5
NCTC 12903; ATCC 27853	RW11	CLIN	79	15.5	10	16
RW109		IND; PC	55	17	14	8
RW110		IND; HC	80	22	14.5	31.5
RW130		IND; HC	27.5[a]	8	7	-
RW131		IND; HC	27.6[a]	8.5	7.5	-
RW138		IND; HC	23.5	11.5	12.5	8.75
RW146		IND; HC	25.5[a]	9.5	9	-
RW149		IND; PC	61.5	8	7	-
ATCC 9027	RW151	CLIN	71.5	31	22.5	15.5
RW172		IND; HC	58	16	8	18
RW176		IND; HC	63.5	40	15	24.5
RW184		IND; HC	-	-	-	-
RW192		IND; HC	32.5	18	9.5	16.5
ATCC 13388; NCTC 8060	RW196	UNKNOWN	64	9.5	-	-
ATCC 15442	RW198	ENV	71.5	8.5	-	-
RW199		IND; MWF	35.5	8.5	8	13
RW200		IND; TC	75	24	60[b]	39.5
RW202		IND; HC	76	25	16	36
RW204		IND; HC	76.5	32	35	38

Footnotes: CF, cystic fibrosis; CLIN, clinical; ENV, environmental; IND, industrial; HC, household cleaner; PC, personal care; MWF, metal working fluid; TC, timber care, '-' designates non motile, and light blue shading indicates highly motile. [a]Strains exhibiting an atypical swimming motility are shown in Figure S2B; [b]A strain exhibiting typical swarming with a diameter of approximately 60 mm is shown in Figure S2A

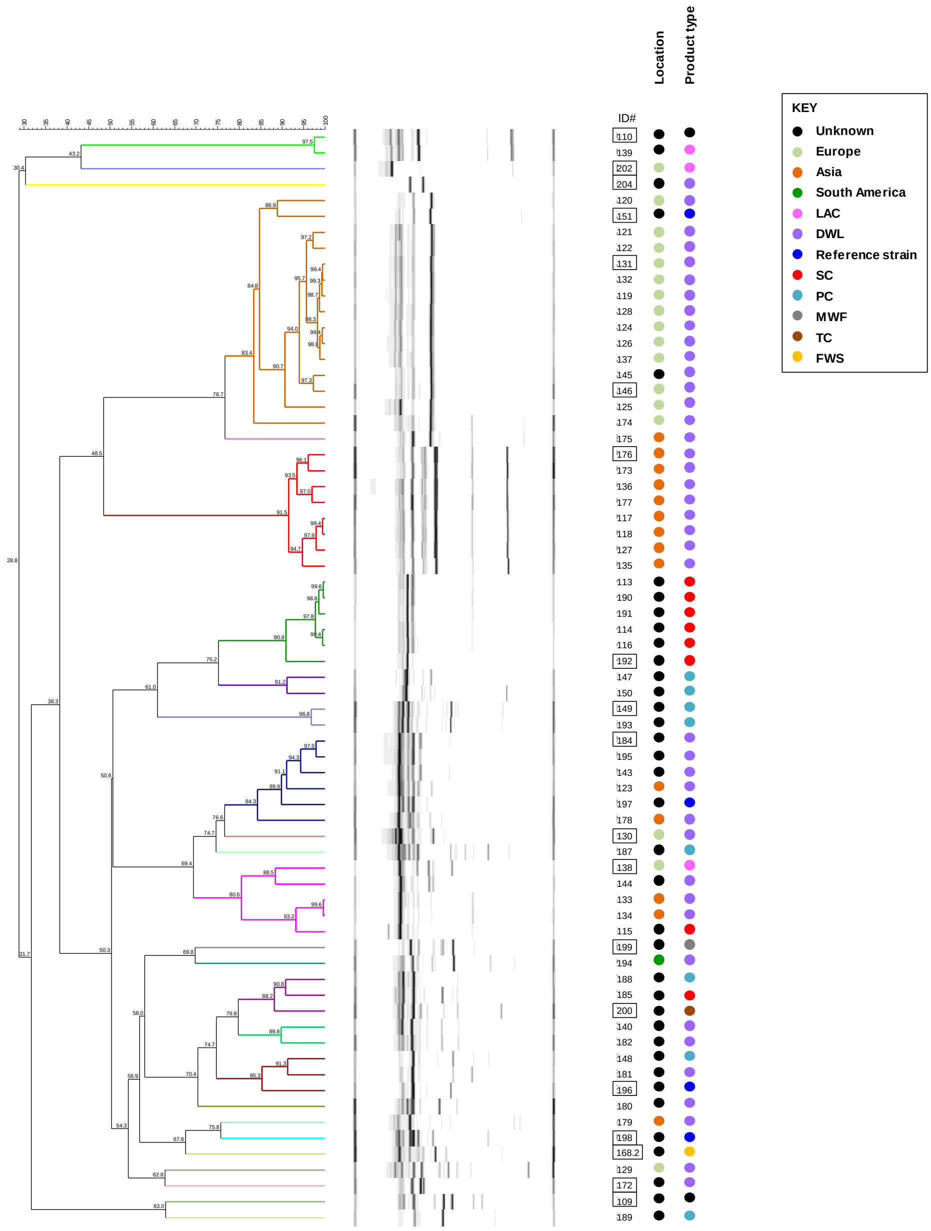

Figure S1. Clustered RAPD-PCR profiles of 69 industrial *P. aeruginosa* isolates. A Pearson correlation similarity coefficient was used to construct a UPGMA dendrogram of the RAPD-PCR profiles. The PCR fingerprint profile of each isolate is shown to the right of the dendrogram, along with the isolate number in the RW collection (ID#; See Table S1 for isolate details) and coloured coded information about isolate provenance. Percentage similarities are indicated by the scale bar and on the branches of the dendrogram. Profiles sharing ≥80% similarity were putatively considered same strain and different strain types are indicated by different coloured branches. Black boxes around ID numbers indicate isolates selected for Clondiag AT typing and genome sequencing. Product type codes are follows: TC, timber care; MWF, metal working fluid; household cleaners (three types DWL, SC and LAC); PC, personal care cosmetic product; and FWS, laundry liquid.

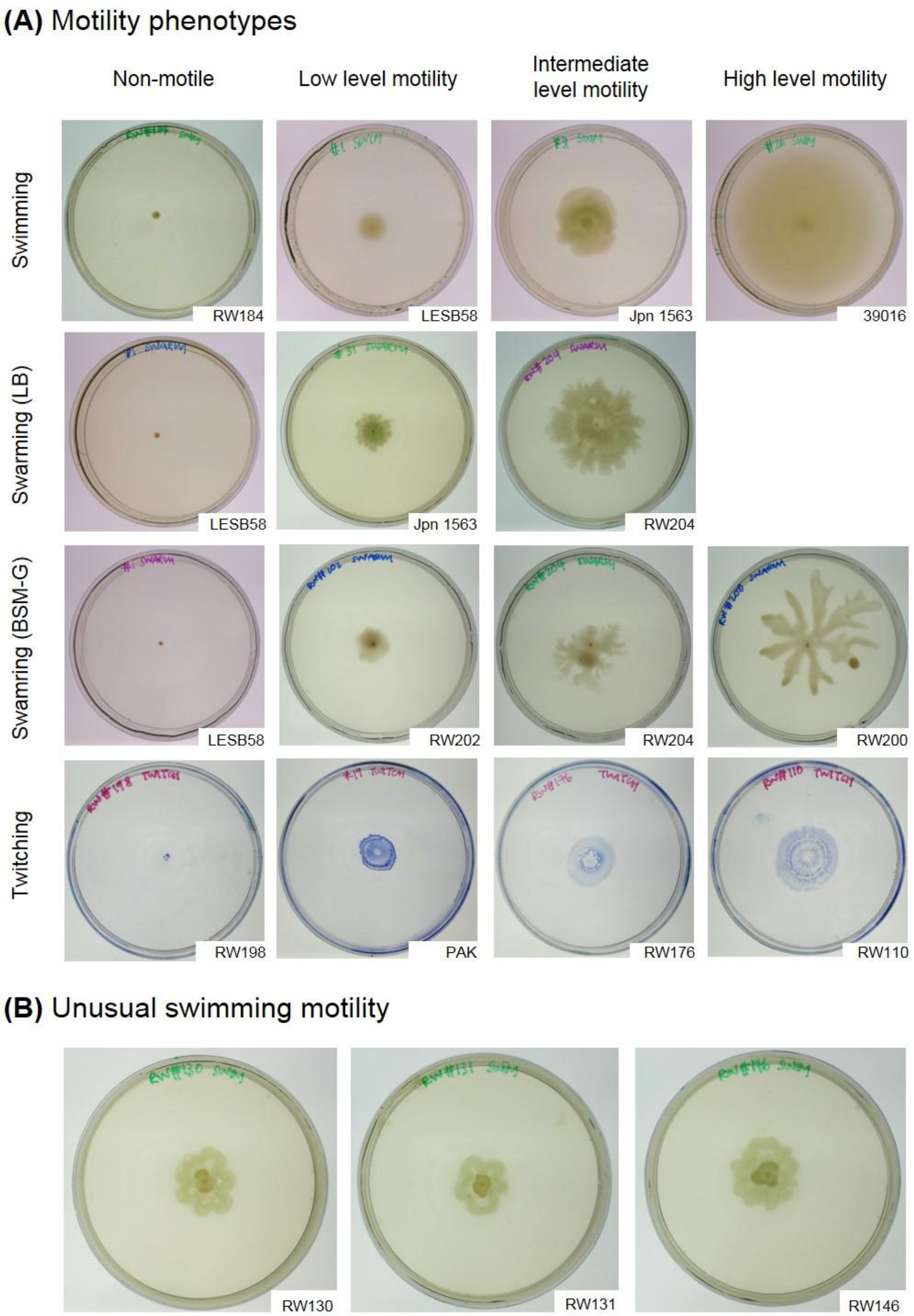

Figure S2. Swimming, swarming and twitching motilities of the *P. aeruginosa* strains. Representative images of the levels of swimming (0.3% LB agar), swarming (0.5% LB and BSM-G agar) and twitching (1% LB agar) motility exhibited are shown in panel A. Results were recorded after 16-18 hours incubation at 30°C (swarming) or 37°C (swimming and twitching). The unusual but consistent swimming motility phenotype of the 3 industrial strains from the same location are shown in panel B. Numerical assessment of the phenotypes is provided in Table S5.

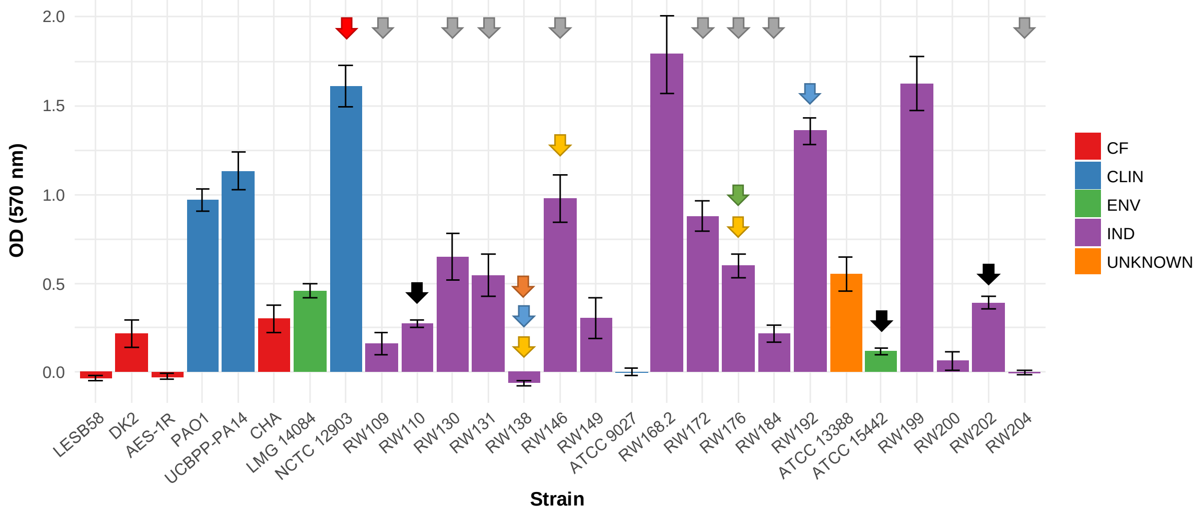

Figure S3. Biofilm formation by *P. aeruginosa* panel and industrial strains. Crystal violet staining of biofilm biomass at 32 hours incubation in TSB medium at 37°C was performed for a subset of 27 *P. aeruginosa* strains, including all 16 industrial strains. Results are presented as mean OD (570 nm) ± standard error and are derived from 3 biological replicates each containing 6 technical replicates. Strains are colour coded to indicate isolation source as cystic fibrosis (CF, red, n=4), clinical (CLIN, blue, n=4), environmental (ENV, green, n=2), industrial (IND, purple, n=16) and unknown (orange, n=1). Industrial strains carrying a megaplasmid are highlighted with grey arrows, high MIT MICs with yellow arrows, high CITMIT MICs with green arrows, high BIT MICs with blue arrows, high PHE MICs with orange arrows, high CHX MICs with black arrows and high BA MICs with red arrows. Strain ATCC 9027 is of clinical origin but had very limited biofilm forming ability (the bar is too small to see the colour).

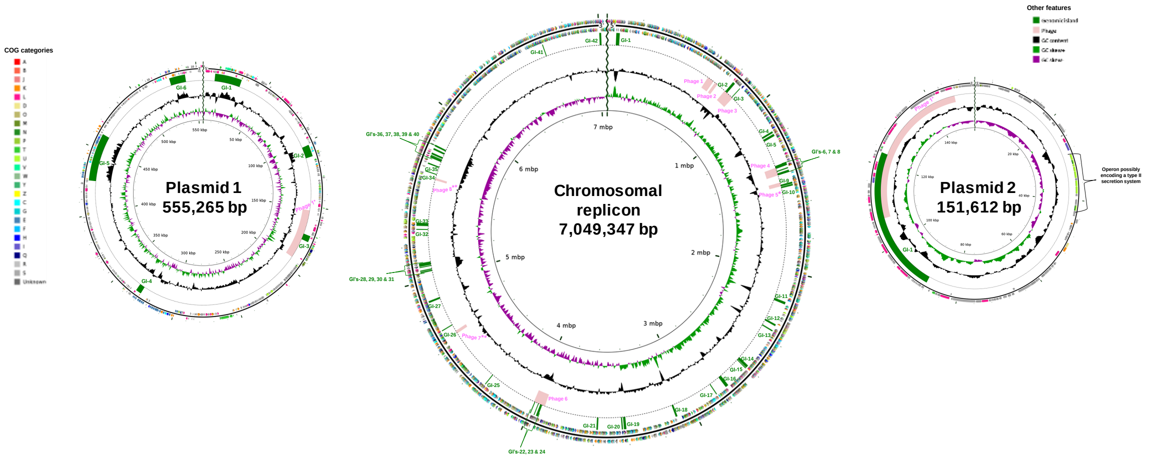

Figure S4. The complete multireplicon genome of industrial *P. aeruginosa* strain RW109. Circular maps of the RW109 replicons are shown with the following tracks (outer to inner rings): (1) predicted CDS on forward strand coloured according to COG categories; (2) predicted CDS on reverse strand coloured according to COG categories; (3) genomic islands (GIs) coloured green; (4) phages coloured light pink (those labelled with * indicate an incomplete phage and those labelled with ** indicate a putative phage identification); (5) GC content (black); (6) positive and negative GC skew (green and purple, respectively); and (7) genome size scales (mbp for the main chromosome and kbp for plasmids 1 and 2; replicons are not drawn to scale).

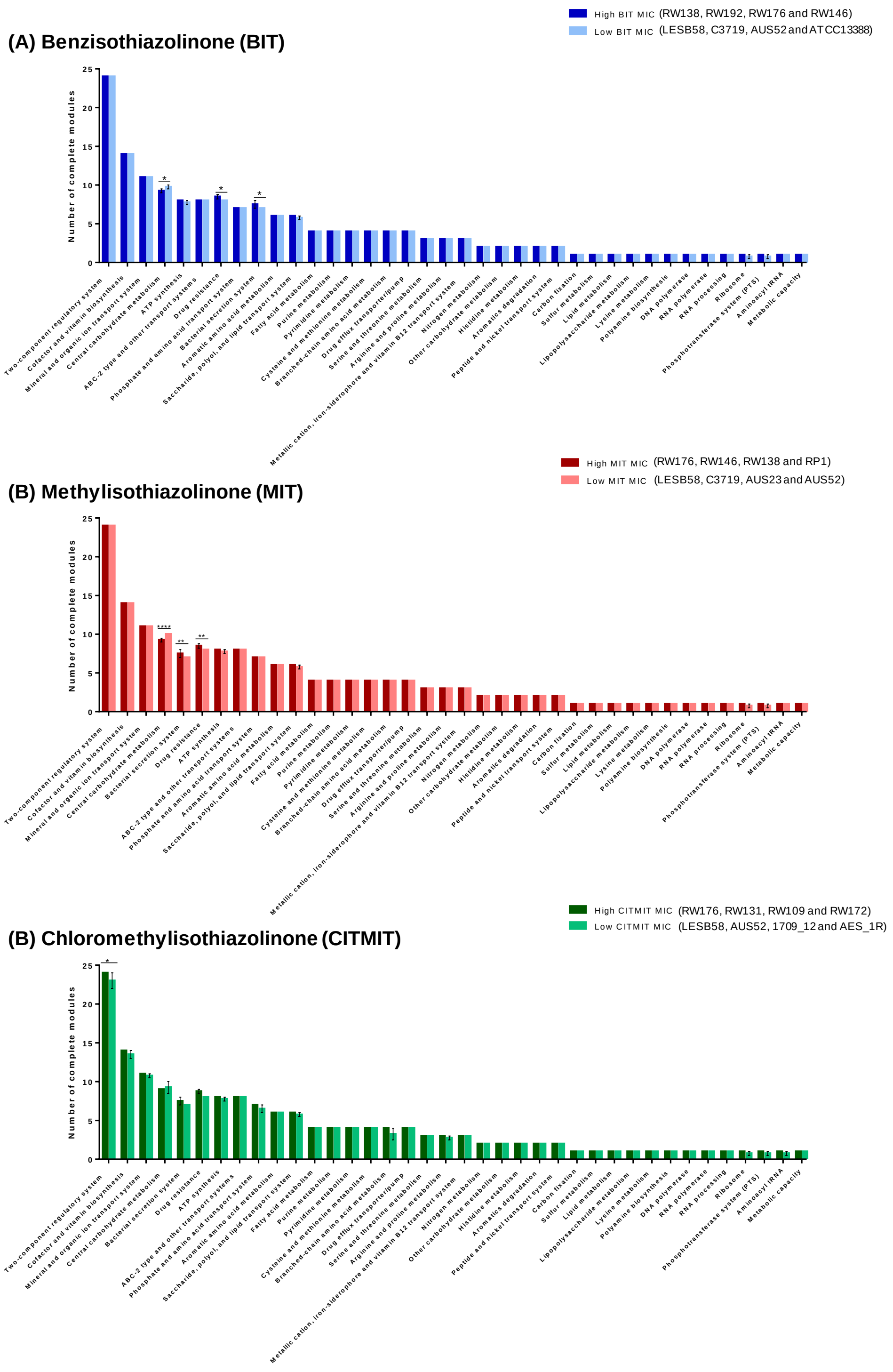

Figure S5. KEGG functional module enrichment analysis in relation to *P. aeruginosa* tolerance of BIT, MIT and CITMIT. Four selected *P. aeruginosa* strains with the highest and four with lowest MIC in relation to each preservative (see top right key for each panel) were compared for the KEGG functional module content (see Supplementary Methods). Significant differences were determined by two-way ANOVA (Sidak's multiple comparisons test) and the results for: (A) BIT, (B) MIT and (C) CITMIT, plotted in the respective panels. For BIT, high MIC strains were enriched for drug resistance (p = 0.0117) and bacterial secretion system (p = 0.0117) modules (panel A); MIT tolerant *P. aeruginosa* strains showed enrichment of exactly the same function modules (drug resistance, p = 0.0061; and bacterial secretion system, p = 0.0061; panel B). Low BIT and MIT MIC strains had an increased number of modules for central carbohydrate metabolism (p = 0.0117 and p = <0.0001, respectively). Two component regulatory system functional modules were significantly enriched in relation to CIT MIT (p = 0.0347).

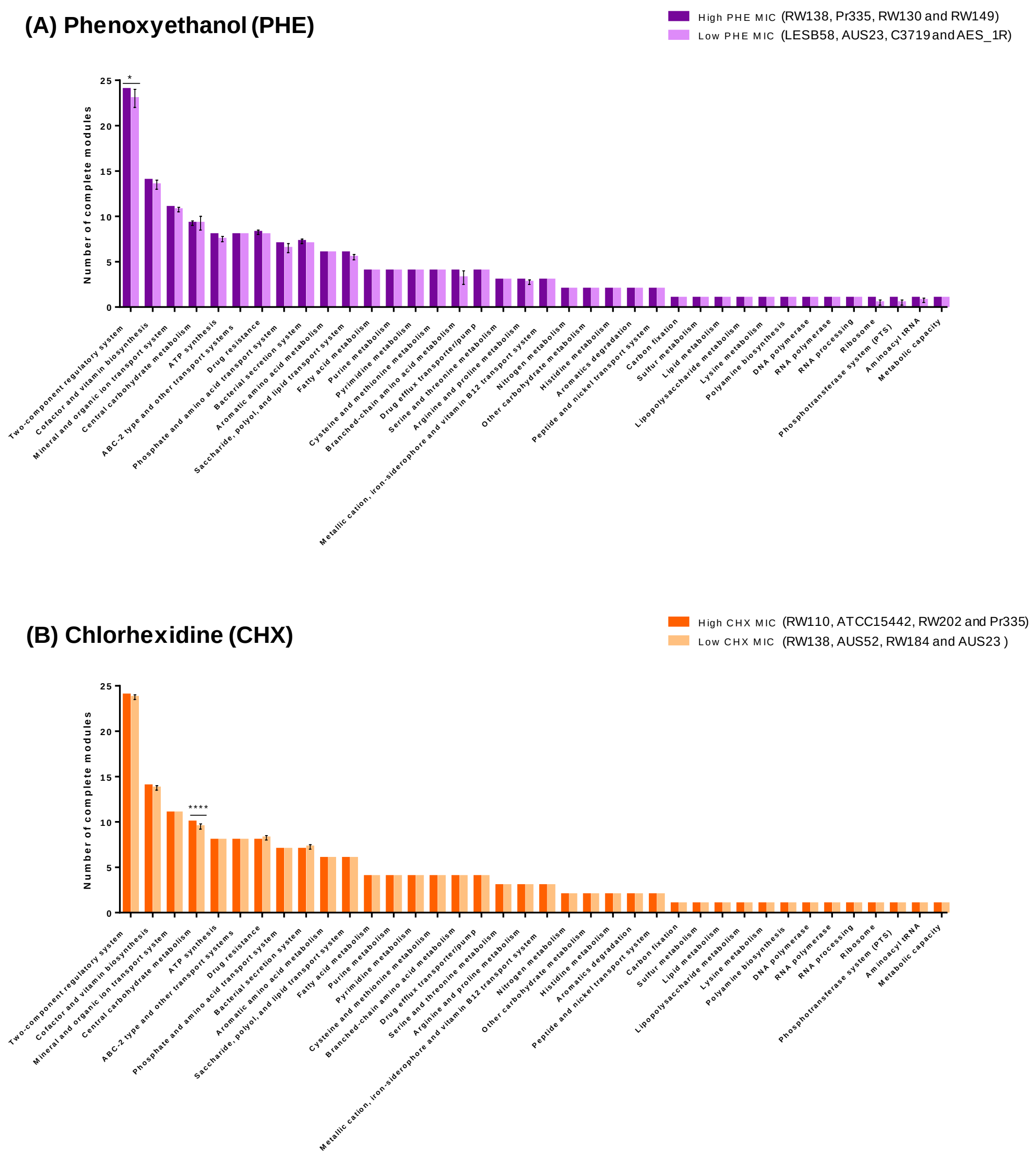

Figure S6. KEGG functional module enrichment analysis in relation to *P. aeruginosa* tolerance of PHE and CHX. Four selected *P. aeruginosa* strains with the highest and four lowest MIC in relation to each preservative (see top right key for each panel) were compared for the KEGG functional module content (see Supplementary Methods). Significant differences were determined by two-way ANOVA (Sidak's multiple comparisons test) and the results for (A) PHE and (B) CHX plotted in the respective panels above. *P. aeruginosa* strains tolerant of PHE showed significant enrichment of two component regulatory system KEGG functional modules (p = 0.0323), and CHX tolerant strains contained more central carbohydrate metabolism modules (p = <0.0001).
